# CRSP8 promotes thyroid cancer progression by antagonizing IKKα-induced cell differentiation

**DOI:** 10.1038/s41418-020-00656-0

**Published:** 2020-11-08

**Authors:** Yina Liao, Yijun Hua, Yizhuo Li, Changlin Zhang, Wendan Yu, Ping Guo, Kun Zou, Wenyang Li, Yao Sun, Ruozhu Wang, Yan Zuo, Silei Sui, Chunfang Tian, Jiaojiao Hao, Manyu Chen, Sheng Hu, Miao Chen, Qian Long, Xiaonan Wang, Lijuan Zou, Fangyun Xie, Wei Guo, Wuguo Deng

**Affiliations:** 1 Institute of Cancer Stem Cells & The First Affiliated Hospital, Dalian Medical University, Dalian, China; 2 Shanghai Center for Thyroid Disease, Shanghai Tenth Peopleʼs Hospital, School of Medicine, Tongji University, Shanghai, China; 3 Sun Yat-sen University Cancer Center; State Key Laboratory of Oncology in South China; Collaborative Innovation Center of Cancer Medicine, Guangzhou, China; 4 The Seventh Affiliated Hospital, Sun Yat-sen University, Shenzhen, China; 5 The Second Affiliated Hospital, Dalian Medical University, Dalian, China

**Keywords:** Oncogenes, Gene regulation

## Abstract

CRSP8 plays an important role in recruiting mediators to genes through direct interaction with various DNA-bound transactivators. In this study, we uncovered the unique function of CRSP8 in suppressing thyroid cancer differentiation and promoting thyroid cancer progression via targeting IKKα signaling. CRSP8 was highly expressed in human thyroid cancer cells and tissues, especially in anaplastic thyroid cancer (ATC). Knockdown of CRSP8 suppressed cell growth, migration, invasion, stemness, and induced apoptosis and differentiation in ATC cells, while its overexpression displayed opposite effects in differentiated thyroid cancer (DTC) cells. Mechanistically, CRSP8 downregulated IKKα expression by binding to the IKKα promoter region (−257 to −143) to negatively regulate its transcription. Knockdown or overexpression of IKKα significantly reversed the expression changes of the differentiation and EMT-related markers and cell growth changes mediated by CRSP8 knockdown or overexpression in ATC or DTC cells. The in vivo study also validated that CRSP8 knockdown inhibited the growth of thyroid cancer by upregulating IKKα signaling in a mouse model of human ATC. Furthermore, we found that CRSP8 regulated the sensitivity of thyroid cancer cells to chemotherapeutics, including cisplatin and epirubicin. Collectively, our results demonstrated that CRSP8 functioned as a modulator of IKKα signaling and a suppressor of thyroid cancer differentiation, suggesting a potential therapeutic strategy for ATC by targeting CRSP8/IKKα pathway.

## Introduction

As one of the most common cancers originated from the endocrine system, the incidence of thyroid cancer has been increasing rapidly worldwide [1]. Approximately 52,070 new cases of thyroid cancer have been diagnosed in the United States in 2019 [2]. Thyroid cancers are derived from two types of endocrine thyroid cells, follicular thyroid cells, and parafollicular C cells. Follicular thyroid cell-derived tumors, including papillary thyroid cancer (PTC), follicular thyroid cancer (FTC), poorly differentiated thyroid cancer (PDTC), and anaplastic thyroid cancer (ATC), account for the majority of thyroid malignancies, while parafollicular C cell-derived medullary thyroid cancer accounts for only a small proportion. Among various subtypes, PDTC and ATC are much less common, aggressive, and lethal with rapid progression, forming the main cause of thyroid cancer-related mortality [3–5]. The mean survival time of ATC is less than 6 months after diagnosis and the 5-year survival rate is only 0–10% [6]. To date, ATC treatment has not been standardized and there is not yet an effective treatment method [7, 8]. Therefore, the mechanisms underlying the regulation of poor differentiation or anaplastic phenotype in PDTC and ATC deserve in-depth investigation to provide more effective therapeutic strategies.

The differentiation status of tumor cells is closely related to their malignancy grade. In general, less differentiation means a greater likelihood of malignant behavior. Therefore, differentiation induction, aiming to reactivate endogenous differentiation programs in cancer cells to recover the maturation process and eliminate tumor phenotypes, such as self-renewal, unlimited proliferation, immunological tolerance, chemoresistance, and so on, has become the first choice of anticancer therapy. Such therapeutic strategies have already been applied in patients with acute promyelocytic leukemia by using all-trans-retinoic acid to induce terminal cell differentiation [9, 10]. Restoring IKKα expression was reported to induce cellular differentiation in nasopharyngeal carcinoma [11]. In addition, the decreased IKKα expression was shown to be associated with poor differentiation and malignancy of human squamous cell carcinoma [12]. Nevertheless, the accurate function and the underlying molecular mechanisms for IKKα in differentiation induction have still been poorly understood.

The CRSP complex, a multi-protein coactivator consisting of at least 25 subunits, is vital for the transcriptional regulation of eukaryotic genes. It functionalizes as a central integrator of transcription via linking activators and the basal transcription machinery [13–15]. CRSP8, as one of these elements, plays an important role in recruiting mediator to genes through direct interactions with various DNA-bound transactivators [16–18]. It is required for SP1-mediated transcriptional activation [19]. Its knockdown, combined with knockdown of other CRSP proteins, significantly impaired viral replication without affecting cell viability [20]. The pro-tumorigenic function of CRSP8 in melanoma was shown in our previous study [21]. However, the association between abnormal CRSP8 expression and cancer progression is rarely reported in other cancer types, including thyroid cancer. Its exact downstream regulatory mechanisms involved in cancer development also remain unclear.

Here, we uncovered the unique function of CRSP8 in suppressing thyroid cancer differentiation and promoting thyroid cancer progression via anchoring at IKKα promoter to transcriptionally repress IKKα expression. CRSP8 was overexpressed in human thyroid cancer cells and tissues, especially in ATC. Its knockdown inhibited growth, migration, invasion and stemness, and induced apoptosis and differentiation in ATC cells, while its overexpression in differentiated thyroid cancer (DTC) caused reverse effects. The downregulation or upregulation of IKKα rescued the above function changes, especially differentiation associated phenotypes in vitro and in vivo. Collectively, our study unveiled that CRSP8 functionalized as a novel pro-oncogenic protein by targeting IKKα in thyroid cancer, and conferred a therapeutic vulnerability for thyroid cancer treatment, especially ATC.

## Materials and methods

### Cell culture and reagents

All cells were kindly provided by Dr. Quentin Liu (Dalian Medical University, Dalian, China). The PTC cells BCPAP and FTC cells FTC-133, and ATC cells ACT-1 were cultured in DMEM medium. The ATC cells THJ-16T, THJ-21T, THJ-29T, and normal thyroid cells Nthy-ori 3-1 were cultured in RPMI 1640 medium. All culture media was supplemented with 10% fetal bovine serum and antibiotics (100 U/ml penicillin and 100 µg/ml streptomycin) and cells were incubated in a humidified atmosphere containing 5% CO_2_ at 37 °C.

Cisplatin (DDP, #P4394) and epirubicin (EPI, #E9406) were purchased from Sigma-Aldrich. QNZ (#S4902), JSH-23 (#S7351) and Z-VAD-FMK (Z-VAD, #S7023) were purchased from Selleck Chemicals.

### Plasmid constructs and transfection

The CRSP8 overexpression vector pcDNA3.1-CRSP8 or control vector pcDNA3.1-LacZ plasmids were designed and synthesized by Addgene (#15424). The plasmids encoding human IKKα were provided by the professor Quentin Liu in Dalian Medical University, Dalian, China. The expression plasmids were transfected into cells with 0.2 or 2 μg for each well of 96-well plates or 6-well plates using Lipofectamine 2000 (Invitrogen) according to the manufacturer’s instructions.

### siRNA design and transfection

The siRNAs targeting CRSP8 (siRNA1: 5′-GCGGACGUGAUAAAUGUCAT T-3′ and 5′-UGACAUUUAUCACGUCCGCTT-3′; siRNA2: 5′-CUGGUUAAGAAG UUACAUATT-3′ and 5′-UAUGUAACUUCUUAACCAGTT-3′), siRNAs targeting IKKα (siRNA1: 5′-GCAGGCUCUUUCAGGGACATT-3′ and 5′-UGUCCCUGAAA GAGCCUGCTT-3′; siRNA2: 5′-CAAAGAAGCUGACAAUACUTT-3′ and 5′-AGU AUUGUCAGCUUCUUUGTT-3′), negative control siRNA (5′-UUCUCCGAACGU GUCACGUTT-3′ and 5′-ACGUGACACGUUCGGAGAATT-3′) were purchased from Shanghai GenePharma Co (Shanghai, China). Cells plated in 96-well plates or 6-well plates were transfected with 0.2 or 2 μg of siRNA for each well using Lipofectamine 2000 according to the manufacturer’s instructions. At 48 h after treatment, RNAi efficiency was determined by real-time RT-PCR and western blot.

### RT-PCR

Total RNA was extracted from cultured cell lines by using Trizol Reagent (TaKaRa Bio) according to the manufacturer’s instructions. cDNA synthesis was performed using TransScript One-Step gDNA Removal and cDNA Synthesis SuperMix (TRAN) according to the protocol described, and RT-PCR was finished by using 2×EasyTaq PCR SuperMix (TRAN) as recommended by the manufacturer. The sequences were as follows: for CRSP8 (forward: 5′-GTGTTCGACTGCCTGAAGG A-3′, reverse: 5′-G GACAAGATTGTGGGCTGA-3′); for IKKα (forward: 5′-TAACC CCTCAAGCATACGCA-3′, reverse: 5′-TTGTGCTGAAGTCTCCCCATC-3′); for NIS (forward: 5′-GGCGTCGCTCCTGTCCAC-3′, reverse: 5′-CGCCCACAAGCAT GACAC-3′); for Tg (forward: 5′-GCGGGAACTGGCTGAGA-3′, reverse: 5′-GTGG GCAGGATGTGGCAAAT-3′); for GAPDH (forward: 5′-AATCCCATCACCATCTT CC-3′, reverse: 5′-C ATCACGCCACAGTTTCC-3′).

### Database analysis

UALCAN analysis (http://ualcan.path.uab.edu/index.html) was used to estimate the expression levels of CRSP8 or SP1 based on cancer stages, sample types, and histological subtype in the Cancer Genome Atlas (TCGA) thyroid cancer datasets. The correlation of CRSP8 and CHUK (IKKα) expression, CRSP8 and SP1 expression, SP1 and CHUK (IKKα) expression in thyroid cancer were analyzed using the cBioPortal for Cancer Genomics database (www.cbioportal.org). The expression levels of CRSP8 in various subtypes of thyroid cancer tissues were identified from Oncomine database (https://www.oncomine.org/resource/login.html), which collected and analyzed data from the cancer genome map of 99 patients. Expression profiles of mRNA data of thyroid cancer were downloaded from Gene Expression Omnibus (GEO) database (http://www.ncbi. nlm.nih.gov/geo/), and dataset GSE53072 was used to analyze the expression of CRSP8 in normal and anaplastic thyroid carcinoma.

### Immunoblotting

Cells were lysed on ice in protein extraction reagent and protein concentration was determined by using BCA protein Assay Kit (SWBio). Equal amounts of cell extracts were subjected to electrophoresis in 10–15% gradient SDS-PAGE gels, and then transferred to polyvinylidene fluoride membranes, and immunoblotted with specific primary antibodies, followed by incubation with HRP-conjugated secondary antibody and finally detected by using ECL (Electro-Chemi-Luminescence) substrates. Quantification and analysis were performed using Image J software. Antibodies against E-cadherin (Proteintech, 20874-1-AP, dilution 1:5000), N-cadherin (Proteintech, 22018-1-AP, dilution 1:2000), Slug (Cell Signaling Technology, #9585, dilution 1:1000), Vimentin (Cell Signaling Technology, #5741, dilution 1:1000), β-catenin (Cell Signaling Technology, #8480, dilution 1:1000), p-AKT (Cell Signaling Technology, #4060, dilution 1:2000), AKT (Cell Signaling Technology, #4691, dilution 1:1000), cleaved caspase-3 (Cell Signaling Technology, #9664, dilution 1:1000), p85 (Cell Signaling Technology, #4257, dilution 1:1000), p-p85 (Cell Signaling Technology, #4228, dilution 1:1000), p110α (Cell Signaling Technology, #4249, dilution 1:1000), Bax (Proteintech, 50599-2-Ig, dilution 1:6000), Bcl-2 (Proteintech, 12789-1-AP, dilution 1:2000), cleaved caspase-9 (Cell Signaling Technology, #7237, dilution 1:1000), CD133 (Proteintech, 18470-1-AP, dilution 1:1000), CD44 (Proteintech, 15675-1-AP, dilution 1:4000), β-actin (Proteintech, 20536-1-AP, dilution 1:3000), MMP-9 (Affinity Biosciences, AF5228, dilution 1:1000), CRSP8 (Santa Cruz, sc-390295, dilution 1:500), IKKα (Cell Signaling Technology, #11930, dilution 1:1000), NIS (Proteintech, 24324-1-AP, dilution 1:1000), Tg (Abcam, ab156008, dilution 1:10000), Bad (Cell Signaling Technology, #9292, dilution 1:1000), Bim (Cell Signaling Technology, #2933, dilution 1:1000), RAD51 (Proteintech, 14961-1-AP, dilution 1:1000), γH2AX (Cell Signaling Technology, #9718, dilution 1:1000), LC3 I/II (Cell Signaling Technology, #12741, dilution 1:1000), p62 (Proteintech, 55274-1-AP, dilution 1:1000), p65 (Cell Signaling Technology, #8242, dilution 1:1000), p-p65 (Cell Signaling Technology, #3033, dilution 1:1000), p50 (Santa Cruz, sc-8414, dilution 1:500), IKKβ (Cell Signaling Technology, #8943, dilution 1:1000), p-IKKα/β (Cell Signaling Technology, #2697, dilution 1:1000), IκBα (Cell Signaling Technology, #4812, dilution 1:1000), p-IκBα (Cell Signaling Technology, #2859, dilution 1:1000) were purchased from the indicated companies.

### MTT assay

Cells plated in 96-well plates (4000–6000 cells/well) were treated with drugs or plasmids or siRNAs. After treatment for the desired time, 10% MTT was added to the cells with continuous culture for another 4 h. Then the absorbance value at OD490 was detected.

### Colony formation assays

Approximately 500–1000 cells were seeded into six-well plates in triplicate and incubated for 10–14 days. Then the cells were washed with PBS and fixed with the mixture (methanol: glacial: acetic = 1:1:8) for 10 min, and stained with 0.1% crystal violet for 15 min. After enough washing by PBS, cells were photographed and the cell colonies that contained more than 50 cells were counted.

### Wound-healing assay

The wound-healing assay was performed to examine the cellular capability of migration. Cells were plated in a six-well plate and grown to nearly 70–80% confluence. Then the cells were treated with CRSP8 siRNAs or plasmids for 12 h and scraped in a straight line to create a “scratch”. The images of the cells at the beginning and at regular intervals during cell migration to close the scratch were captured and compared through quantifying the migration rate of the cells.

### Transwell invasion assay

The transwell invasion assay was performed using 24-well chemotaxis chambers (Corning, USA). Cells seeded in six-well plates were transfected with CRSP8 siRNAs or its overexpression plasmids. After 48 h, cells were collected, resuspended in serum-free medium, counted, and 1 × 10^4^ cells from each group were added to each upper chamber, the inner bottom of which was pre-coated with Matrigel Matrix (Corning, USA) and air dried naturally. 500 μl medium containing 20% fetal bovine serum was filled in the lower chambers. After being incubated at 37 °C for 48 h, the cells located at the underside of the filter were washed twice with PBS, fixed with 4% paraformaldehyde for 10 min and stained with 0.1% crystal violet for 15 min. Images of the cells were taken by inverted microscope, and the invasiveness of cells was defined by the average number of cells in three randomly selected microscopic fields.

### Apoptosis assay

Apoptosis was measured based on FACS analysis by using FITC-AV/PI staining.

Cells with indicated treatment were collected, washed twice with cold PBS, resuspended with cold binding buffer, and subsequently stained with AnnexinV-FITC and Propidium Iodide. Stained cells were then analyzed by using FACS Accuri C6.

### Acridine orange/ethidium bromide (AO/EB) fluorescence staining

The cells were grown on chamber slides and treated with indicated siRNAs. After 48 h, cells were washed by PBS to remove detached cells, and then fixed by 95% ethanol for 15 min. After slightly drying the cells, 10 μl of AO/EB (50 μg/ml) was gently mixed and added into each well, and then the cells were photographed by Leica DM 14000B microscope equipped with digital camera. The cells with apoptosis displayed much brighter orange-red fluorescence accumulation in the nucleus as more EB passed through the damaged cell membrane and embedded in nuclear DNA. Apoptotic cell counting was processed with Image J software.

### Tumorsphere culture

Thyroid cancer cells with indicated treatment were digested into single cells and were seeded in 35 mm ultralow attachment dishes (BIOFIL, 2000 cells/dish) with continuous culture in serum-free DMEM/F12 medium (HyClone) containing B27 supplement (Gibco), bFGF (20 ng/ml), and EGF (20 ng/ml) for 2 weeks. Then the pictures of the formed tumorspheres were taken by inverted microscope (Leica).

### Confocal immunofluorescence assay

The cells were dropped to the cover glass in each well of six-well plates. After cultured for the desired time, the cells were fixed by 4% paraformaldehyde for 30 min, permeabilized by 0.2% TritonX-100 reagent for 2–5 min, blocked by 10% BSA (bovine serum albumin) diluted in PBS for 30 min, and then incubated overnight with the primary antibody against p50 (Santa Cruz, sc-8414, dilution 1:200) or p65 (Cell Signaling Technology, #8242, dilution 1:400) diluted in PBS containing 1% BSA. After washing with PBS, the slides were incubated with secondary antibodies conjugated with fluorescein isothiocyanate and rhodamine for 1 h in the dark room. Finally, after washing with PBS, the slides were stained with DAPI (Sigma-Aldrich) and anti-fade reagent. Then the protein expression was observed and photographed by using Leica DM14000B confocal laser scanning microscope.

### Luciferase reporter assay

The fragments of the IKKα promoter (−843 to +69, −540 to +69, −257 to +69, −143 to +69) were amplified by PCR and inserted between the HindІІІ and KpnI sites of the firefly luciferase vector pGL3-Basic (Promega Corp, Madison, WI), and Renilla luciferase control reporter vector pRL-TK was used as a control. Cells were seeded in six-well plates. On the next day, the cells were respectively transfected with CRSP8 specific siRNAs or overexpression plasmids. After 24 h, IKKα promoter-driven luciferase plasmids and Renilla luciferase vectors were also co-transfected into the above cells. 24 h after transfection, cells were harvested, and dual-luciferase assay was performed using the Dual-Luciferase^®^ Reporter Assay System (Promega, Madison, WI).

### Pull-down assay

400 μg nuclear proteins were mixed with 0.5 μg double-strand biotinylated IKKα promoter probe or nonspecific probe and 45 μl streptavidin agarose beads in 500 μl prepared PBSI buffer containing 0.5 mM PMSF, 10 mM NaF, 25 mM β-glycerophosphate, then gently rotated at RT overnight. The beads were centrifuged, washed with PBSI buffer twice, and then were resuspended with loading buffer and boiled at 100 °C for 10 min. The supernatant was analyzed by western blot.

### Chromatin immunoprecipitation (ChIP) assay

The cells were “fixed” with 1% formaldehyde, scrapped, collected, and sonicated on ice to shear the DNA into the fragments from 100 to 500 bp. A small portion of the cell lysate was used as the DNA input control, and the remaining total lysate was subjected to immunoprecipitations with anti-CRSP8, RNA Pol II, or IgG antibody. The immunoprecipitated DNA was subjected to PCR to amplify a 114 bp fragment of IKKα promoter. The PCR products were then resolved electrophoretically on a 2% agarose gel and visualized by ethidium bromide staining. The primers used for PCR reaction was as following: 5′-AATACAGGAGAGACTGGGCTGCTTT-3′ and 5′-GGGAGGGCTGAACGGAACCACAATG-3′.

### Immunohistochemistry staining

Immunohistochemical (IHC) staining was performed according to the DAB (3, 3-diaminobenzidine) Kit (Origene, China) and respectively applied to tissue microarrays and subcutaneous tumor tissue of nude mice. Tissue microarray (Core diameter 2.0 mm) used for immunostaining analysis of CRSP8 and IKKα was purchased from Outdo Biotech Company (Shanghai, China). The microarray consisted of 12 DTC tissues, 5 PDTC/ATC tissues, and 12 normal thyroid tissues. The subcutaneous tumor tissues of nude mice used for immunostaining analysis were respectively from four different treatment groups with four mice in each group. Briefly, slides were deparaffinized, rehydrated, and then immersed in a target retrieval solution (pH 6; Dako Cytomation) and boiled at medium baking temperature for three times with 10 min once in a microwave. After blocking the slides with 3% BSA, the sections were then incubated with indicated primary antibody against CRSP8 (dilution 1:50), β-catenin (dilution 1:200), IKKα (dilution 1:100), Vimentin (dilution 1:100), Ki67 (Proteintech, 27309-1-AP, dilution 1:200). For negative controls, the primary antibody was excluded. Then, the sections were incubated with biotinylated secondary antibody followed by adding horseradish peroxidase-conjugated streptavidin. The specimens were counterstained with hematoxylin and the target-positive cells were counted in 3–4 different fields and photographed using an Olympus microscope (Model BX40F4, Tokyo, Japan). The slides were scored independently based on the staining color depth. (−) stands for no yellow or light yellow; (+) stands for light yellow; (++) stands for medium or deep yellow and (+++) stands for deepest yellow even to brown. (−) and (+) represented the low expression group, (++) and (+++) represented the high expression group.

### In vivo tumor model and tissue processing

All animal maintenance and operational procedures were carried in accordance with the animal license protocol approval of Animal Care and Ethics Committee of Dalian Medical University. Four- to six-week-old BALB/c nude male mice were purchased from Beijing Vital River Laboratory Animal Technology Co., Ltd and were randomized for shRNA or siRNA treatment groups. For shRNA treatment, thyroid cancer cells, ACT-1, with stable knockdown of CRSP8 and/or IKKα were firstly established by lentivirus infection encapsulating CRSP8 shRNA and/or IKKα shRNA or negative control shRNA (shCtrl) obtained from GeneCopoeia (Rockville, MD). Then the mice were randomly divided into four groups (4 mice per group): CRSP8 shCtrl + IKKα shCtrl, CRSP8 shRNA + IKKα shCtrl, CRSP8 shCtrl + IKKα shRNA, CRSP8 shRNA + IKKα shRNA, and the established stable cells (1 × 10^7^ suspended in 100 μl PBS) were injected subcutaneously into the right flank of each mouse respectively. For siRNA treatment, ACT-1 cells (1 × 10^7^ in 100 μl PBS) were injected subcutaneously into the left flank of each mouse. When the formed tumor reached 100 mm^3^, the mice were divided randomly into four groups (5 mice per group): CRSP8 siCtrl, CRSP8 siRNA, CRSP8 siRNA+ IKKα siCtrl, CRSP8 siRNA+ IKKα siRNA, and siRNAs conjugated by DC nanoparticles were injected intratumorally twice weekly with 10 μg for each siRNA at a time for each mouse. Tumors were measured perpendicular dimensions using calipers, volumes were estimated using the formula (a^2^ × b)/2, where a is the shorter one of the two dimensions and b is the longer one. At the final time point, the mice were sacrificed and the tumors were removed, weighed, and partial of them in each group were quickly frozened in liquid nitrogen for WB and RT-PCR analysis, while the rest of them were then fixed in 10% formalin used for the IHC experiment.

### Statistical analysis

Data are represented as mean ± standard deviation (SD). To compare the statistical differences, SPSS software (version 11.0, Chicago, IL, USA) or Graph Pad Prism software (San Diego, CA, USA) was used by two-tailed Student’s *t* test or one-way ANOVA as approximate. *P* < 0.05 was considered to be a statistically significant difference.

## Results

### CRSP8 was highly expressed in human thyroid cancer, especially in ATC

**Fig. 1:**
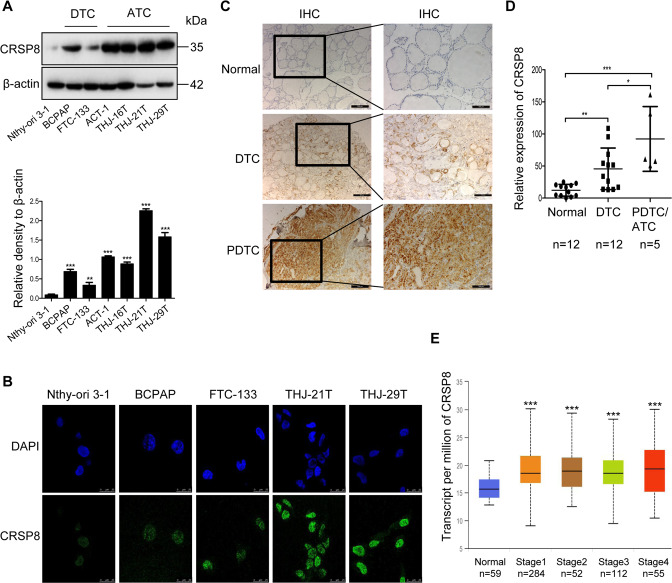
The high expression of CRSP8 in thyroid cancer cells and tissues, especially in ATC. **A** The protein levels of CRSP8 in human normal thyroid cells, DTC cells, and ATC cells were detected by western blot. **B** The immunofluorescence analysis of the expression and localization of CRSP8 in human normal thyroid cells, DTC cells, and ATC cells. Scale bars, 25 μm. **C** CRSP8 expression in normal thyroid tissues, DTC tissues, and PDTC tissues was observed by immunohistochemistry staining. Scale bars, 200, 100 μm. **D** Relative expression of CRSP8 by tissue type was shown in the scatter plot. 12 normal, 12 DTC, and 5 PDTC/ATC tissue samples were analyzed. **E** Expression of CRSP8 in thyroid cancer based on individual cancer stages from UALCAN analysis. The data represent the mean ± SD of three independent experiments, and the level of significance was indicated by ***P < 0.001, **P < 0.01, *P < 0.05. ATC anaplastic thyroid cancer, DTC differentiated thyroid cancer, PDTC poorly differentiated thyroid cancer.

To investigate the role of CRSP8 in thyroid cancer progression, we first analyzed its expression in human normal thyroid cells, DTC cells, and ATC cells. Compared to normal thyroid cells, CRSP8 was highly expressed in human thyroid cancer cells, especially in ATC, and mainly located in the nucleus (Fig. 1A, B). IHC staining based on human thyroid cancer tissue microarrays similarly indicated that thyroid cancer tissues, especially PDTC/ATC tissues, exhibited relatively strong staining for CRSP8, and showed clear CRSP8 nuclear staining in PDTC tissues (Fig. 1C, D).

To evaluate the relationship between CRSP8 expression and the clinicopathologic characteristics, we analyzed thyroid cancer datasets in UALCAN, Oncomine, and GEO. The high expression of CRSP8 was shown to be significantly correlated with the worse status of clinical TNM stages (Fig. 1E). Compared with normal tissues, its expression was increased in primary tumors (Supplementary Fig. S1A). Moreover, a statistically significant overexpression of CRSP8 was found in DTC and ATC (Supplementary Fig. S1B–D). Collectively, these results demonstrated the high expression of CRSP8 in thyroid cancer, especially in ATC, and also suggested its potential tumor-promoting effect in thyroid cancer development.

### CRSP8 promoted the growth of thyroid cancer cells

**Fig. 2:**
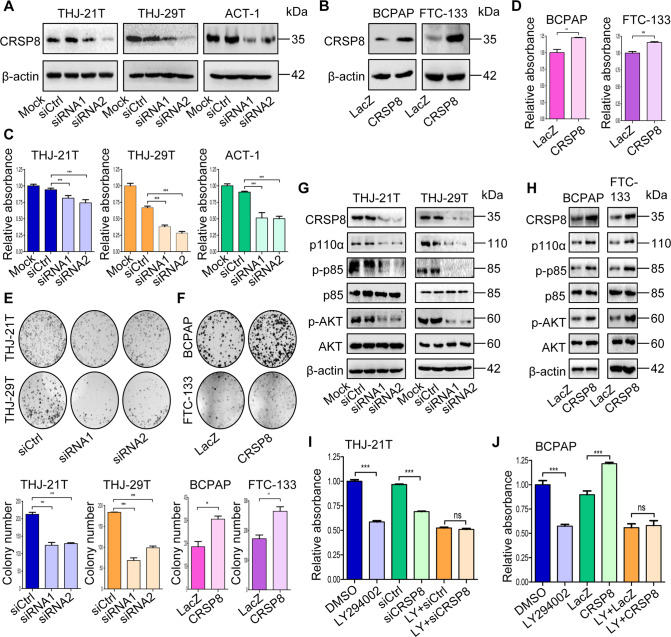
CRSP8 promoted the growth of thyroid cancer cells partially by activating PI3K/AKT signaling pathway. **A**–**H** Thyroid cancer cells were transfected with CRSP8 specific siRNAs or its overexpression plasmids, 48 h after transfection. **A**, **B** CRSP8 expression was analyzed by western blot; **C**, **D** cell viability was tested by MTT assay; **E**, **F** colony formation assay was performed and colony number was counted; **G**, **H** the protein levels of CRSP8, p110α, p-p85, p85, p-AKT, AKT were detected by western blot. **I**, **J** THJ-21T and BCPAP cells were pretreated with a PI3K inhibitor LY294002 (50 μM) for 4 h, and then transfected with CRSP8 specific siRNAs or overexpression plasmids. After 48 h, cell viability was determined by MTT analysis. The data represent the mean ± SD of three independent experiments, and the level of significance was indicated by ***P < 0.001, **P < 0.01, *P < 0.05, ns nonsignificant (*p* > 0.05). LY: LY294002.

Next, we observed the effect of CRSP8 on thyroid cancer cell growth. Silencing of CRSP8 inhibited cell growth and colony formation activities of ATC cells (Fig. 2A, C, E). By contrast, its overexpression in DTC cells showed the opposite effects (Fig. 2B, D, F). These results together indicated that CRSP8 was involved in promoting thyroid cancer cell growth. Most likely, CRSP8 owned a combined effect on thyroid cancer cell proliferation and cell viability.

Given the common and crucial role of PI3K/AKT signaling in mediating cancer cell growth, we then evaluated the effect of CRSP8 expression on this pathway. CRSP8 silencing decreased the expression of p110α, phosphorylated p85, and AKT, while its overexpression increased their expression (Fig. 2G, H). Next, we pretreated cells with LY294002, a highly selective inhibitor of PI3K, before knocking down or overexpressing CRSP8. LY294002 treatment alone significantly inhibited cell growth, whereas CRSP8-siRNA treatment did not obviously synergize with such suppression (Fig. 2I, Supplementary Fig. S2A). Likewise, CRSP8 overexpression did not significantly rescue the growth inhibition caused by LY294002 treatment (Fig. 2J, Supplementary Fig. S2B). Thus, these results provided evidence supporting that PI3K/AKT pathway plays a significant role in CRSP8-mediated growth control, in other words, CRSP8 promotes cell growth, at least partially, by activating PI3K/AKT signaling.

### CRSP8 regulated the migration, invasion, and apoptosis of thyroid cancer cells

**Fig. 3:**
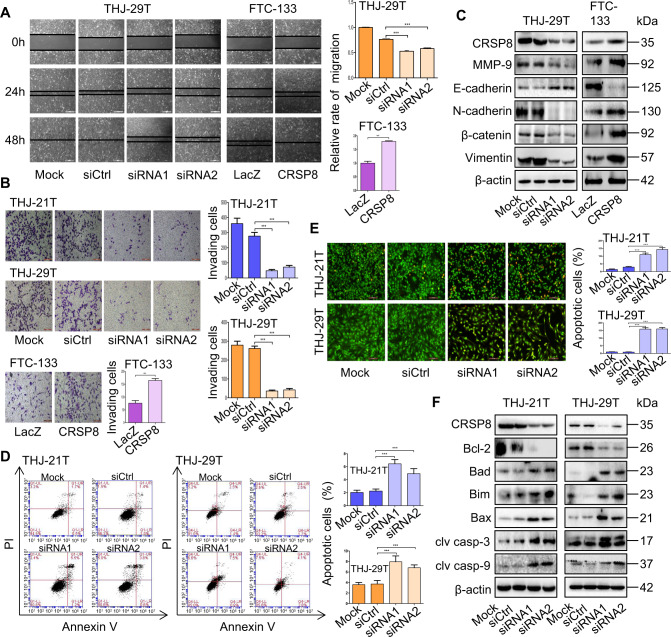
Regulation of CRSP8 on migration, invasion, and apoptosis of thyroid cancer cells. **A** Cell migration and **B** cell invasion were analyzed in thyroid cancer cells transfected with CRSP8 specific siRNAs or its overexpression plasmids, and the relative rate of migration was calculated, the number of invading cells was counted. **C** Western blot analysis of the expression of MMP-9, EMT markers (E-cadherin, N-cadherin, β-catenin, Vimentin) in THJ-29T and FTC-133 cells following CRSP8 silencing or overexpression. THJ-21T and THJ-29T cells were treated with CRSP8 specific siRNAs for 48 h, then **D** FACS analysis and **E** acridine orange/ethidium bromide fluorescence staining were performed, and the apoptotic cell ratios were counted respectively. **F** Western blot analysis of the expression of Bcl-2, Bad, Bim, Bax, cleaved caspase-3, and cleaved caspase-9 proteins in thyroid cancer cells following CRSP8 knockdown. The data represent the mean ± SD of at least three independent experiments (four independent experiments in 3D), and the level of significance was indicated by ***P < 0.001.

The role of CRSP8 on thyroid cancer progression was then assessed by observing its effects on cell migration, invasion, and apoptosis. CRSP8 knockdown significantly suppressed cell migration and invasion, while its overexpression caused the opposite effects (Fig. 3A, B). As epithelial to mesenchymal transition (EMT) process was crucial for tumor invasion and metastasis [22, 23], we then observed the effect of CRSP8 expression on this process in thyroid cancer cells. CRSP8 silencing reduced the expression of EMT inducer (MMP-9), mesenchymal markers (N-cadherin, β-catenin, and Vimentin) and increased the expression of epithelial marker (E-cadherin), while its overexpression produced the opposite effects (Fig. 3C). Collectively, these results supported the promoting role of CRSP8 in thyroid cancer cell metastasis.

To determine whether the inhibition of growth induced by CRSP8 knockdown was realized via apoptosis induction, we next analyzed the effect of CRSP8 knockdown on cell apoptosis. FACS analysis and AO/EB staining indicated that CRSP8 knockdown increased the apoptosis in THJ-21T and THJ-29T cells, leading to more apoptotic cell populations (Fig. 3D, E). In addition, the expression of anti-apoptotic protein Bcl-2 was reduced, but the expression of pro-apoptotic proteins Bad, Bim, Bax, Cleaved Casp-3 and Cleaved Casp-9 were increased upon CRSP8 silencing (Fig. 3F). Next, we pretreated cells with Z-VAD-FMK (abbreviated to Z-VAD), a pan-caspase inhibitor, before knocking down CRSP8. The results showed Z-VAD treatment suppressed the apoptosis initiation caused by CRSP8 knockdown, confirming the presence of caspase-dependent apoptosis upon CRSP8 knockdown (Supplementary Fig. S3A, B). Considering apoptosis and autophagy are two main modes of programmed cell death, we next investigated the regulation of CRSP8 on autophagy in thyroid cancer cells. CRSP8 knockdown decreased LC3-II expression, but increased p62 expression, indicating the inhibition of cell autophagy by CRSP8 silencing, while CRSP8 overexpression showed exactly opposite effects (Supplementary Fig. S3C). Collectively, these data demonstrated CRSP8 knockdown indeed induced cell apoptosis in a caspase-dependent manner, and such cell death induction was not associated with an increased autophagic cell stress response. Considering apoptosis induction was not as significant as cell proliferation and cell viability inhibition upon CRSP8 knockdown, these results also implied the cellular growth inhibition caused by CRSP8 silencing was mainly realized via cell proliferation and viability blockade, but partially by apoptosis induction.

To exclude the possible off-target effects of CRSP8 siRNA and also further confirm the proliferative role of CRSP8 in thyroid cancer cells, we re-transfected CRSP8-silenced cells with CRSP8 overexpression plasmids (Supplementary Fig. S4A). The results showed CRSP8 re-expression restored the decreased cell growth and invasive ability to almost baseline levels in ATC cells (Supplementary Fig. S4B, C), suggesting CRSP8 indeed drives thyroid cancer cell survival.

### CRSP8 suppressed tumor cell differentiation and downregulated IKKα expression in thyroid cancer cells and tissues

**Fig. 4:**
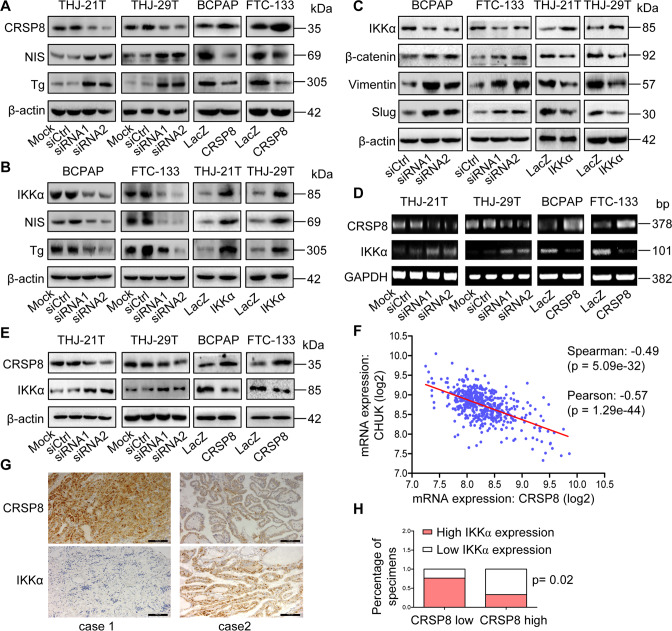
CRSP8 regulated the differentiation of thyroid cancer cells and the negative regulatory effect of CRSP8 on IKKα in thyroid cancer cells and tissues. **A** Western blot analysis of CRSP8, NIS, and Tg expression in thyroid cancer cells transfected with CRSP8 specific siRNAs or its overexpression plasmids. **B** Western blot analysis of IKKα, NIS, and Tg expression in thyroid cancer cells treated with IKKα specific siRNAs or its overexpression plasmids. **C** Western blot analysis of the expression of EMT markers (β-catenin, Vimentin, and Slug) in thyroid cancer cells following IKKα knockdown or overexpression. **D** RT-PCR analysis and **E** western blot analysis of CRSP8, IKKα expression in thyroid cancer cells following CRSP8 knockdown or overexpression. **F** The correlation between CRSP8 and IKKα expression was detected using cBioPortal database. **G** The representative images for CRSP8 and IKKα expression by IHC analysis in human thyroid cancer tissue microarrays. Scale bars, 100 μm. **H** Percentages of specimens with either low or high CRSP8 expression relative to IKKα level.

Considering that CRSP8 was highly expressed in ATC and relative lowly expressed in DTC, we deduced that CRSP8 expression might affect thyroid cancer cell differentiation. Consistently, gene set enrichment analysis from TCGA database suggested that low expression of CRSP8 led to the enrichment of proteins associated with epidermal cell differentiation (Supplementary Fig. S5A). Furthermore, the expression of sodium–iodide symporter (NIS) and thyroglobulin (Tg), the two key markers associated with thyroid cancer differentiation, were significantly increased upon CRSP8 knockdown, but decreased when CRSP8 was overexpressed (Fig. 4A, Supplementary Fig. S5B). Given the close correlation between cellular differentiation status and stem-like traits, we then evaluated the effect of CRSP8 on stemness maintenance of thyroid cancer. CRSP8 silencing significantly suppressed tumorsphere formation ability and expression levels of cancer stemness-related markers, including CD133 and CD44, while its overexpression caused the opposite effects (Supplementary Fig. S5C, D). All these findings collectively demonstrate the crucial role of CRSP8 in the dedifferentiation and stemness maintenance of thyroid cancer cells.

Recent studies have shown the role of IKKα in inducing tumor cell differentiation and reducing tumorigenicity of nasopharyngeal carcinoma independent of its common kinase activity involved in NF-κB activation [11]. Thus, most likely, IKKα similarly regulates thyroid cancer cell differentiation. To explore this, we changed IKKα expression in thyroid cancer cells and determined the expression of NIS and Tg. IKKα silencing reduced NIS and Tg expression, but its overexpression caused the opposite effects (Fig. 4B, Supplementary Fig. S6A), indicating that IKKα indeed regulated the differentiation of thyroid cancer cells. In addition, since EMT was closely associated with cancer stem cell differentiation [24, 25], we then studied the effect of IKKα on EMT. The expression of β-catenin, Vimentin, and Slug were upregulated in cells with IKKα silencing, whereas downregulated in cells with IKKα overexpression (Fig. 4C), suggesting the differentiation promotion mediated by IKKα in thyroid cancer cells was accompanied by the inhibition of the EMT process.

Considering the classic role of IKKα in NF-κB pathway, we further evaluated the dependence of IKKα on this pathway in mediating thyroid cancer cell differentiation. Knockdown or overexpression of IKKα in thyroid cancer cells did not change the nuclear translocation of p65/p50 (Supplementary Fig. S6B, C), and the expression of NIS and Tg regulated by IKKα were not affected when NF-κB signaling was inhibited by treatment with QNZ or JSH-23, which mediated the downregulation of phospho-p65 (Supplementary Fig. S6D, E). Since IKKα inhibited tumor cell apoptosis by activating NF-κB signaling [26], we then investigated whether apoptosis inhibition could rescue the differentiation loss in thyroid cancer cells mediated by IKKα knockdown. Treatment with Z-VAD to inhibit apoptosis cannot significantly reverse the reduced expression of NIS and Tg caused by IKKα knockdown (Supplementary Fig. S6F), suggesting that the regulation of thyroid cancer cell differentiation mediated by IKKα was independent of its inhibition on apoptosis. Together, these data implicated IKKα promoted thyroid cancer cell differentiation bypass its activation on NF-κB signaling.

Since both CRSP8 and IKKα were associated with thyroid cancer differentiation, we next investigated their correlation. CRSP8 knockdown increased IKKα expression at both mRNA and protein levels, while its overexpression decreased IKKα expression (Fig. 4D, E). Such negative correlation between CRSP8 and IKKα expression was also verified in unmanipulated thyroid cancer cells (Supplementary Fig. S7). Furthermore, the data from cBioPortal database also showed that CRSP8 expression was inversely correlated with IKKα expression (Fig. 4F). The immunohistochemistry staining based on human thyroid cancer tissue microarrays also showed the inverse correlation between CRSP8 and IKKα expression (Fig. 4G, H). These data demonstrated the negative regulation of IKKα by CRSP8 in thyroid cancer and the possibility of IKKα functionalizing as the direct downstream effector of CRSP8 in mediating thyroid cancer differentiation.

### CRSP8 dedifferentiated thyroid cancer cells via transcriptionally suppressing IKKα

**Fig. 5:**
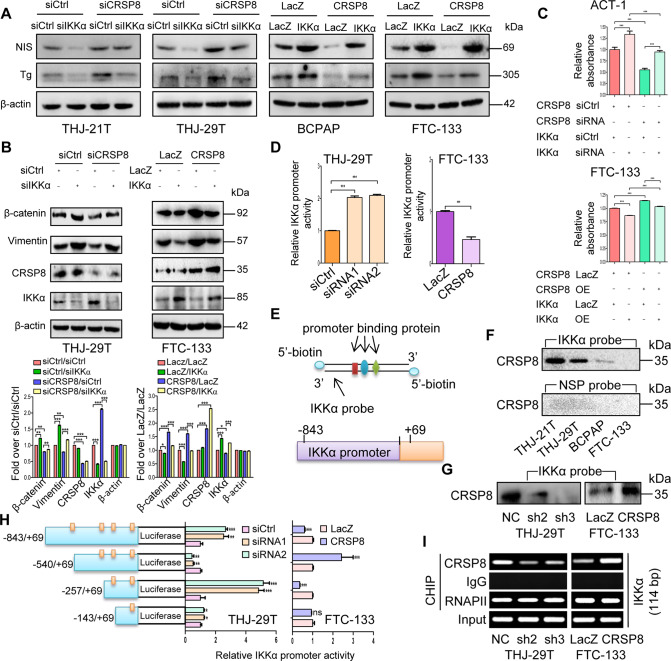
CRSP8 promoted thyroid cancer cell dedifferentiation via transcriptionally regulating IKKα expression. **A**–**C** Anaplastic thyroid cancer cells were transfected with CRSP8 siCtrl or specific siRNAs overnight prior to transfection with IKKα siCtrl or specific siRNAs, while differentiated thyroid cancer cells were treated with control vector or CRSP8 overexpression plasmids overnight and then treated with IKKα control vector or overexpression plasmids. 48 h after treatment, **A** the expression of NIS and Tg were detected by western blot; **B** the expression of EMT-related markers (β-catenin and Vimentin) were analyzed by western blot; **C** cell viability was measured by MTT assay. **D** Dual-luciferase reporter assays were performed to determine relative IKKα promoter activity in CRSP8 knocked down or overexpressed cells. **E** The IKKα promoter probe and structure. **F** Pull-down assay was performed to test the binding of CRSP8 at IKKα promoter in different thyroid cancer cells. NSP nonspecific probe. **G** Pull-down assay was performed to test the binding of CRSP8 to IKKα promoter following CRSP8 silencing or overexpression. **H** Relative activities of different IKKα promoter fragments were detected in thyroid cancer cells using dual-luciferase reporter assays under different expression levels of CRSP8. **I** ChIP assay was performed to test the binding of CRSP8 at the −257 to −143 sites of IKKα promoter. RNA Pol II was used as the positive control, IgG was used as the negative control. The data represent the mean ± SD of three independent experiments, and the level of significance was indicated by ***P < 0.001, **P < 0.01, *P < 0.05.

To confirm whether IKKα was involved in CRSP8-mediated regulation of thyroid cancer cell differentiation, we silenced or overexpressed IKKα in cells with CRSP8 silencing or overexpression, respectively, and observed the cellular phenotype changes. The increased expression of NIS and Tg, the decreased expression of β-catenin and Vimentin, and the reduced cell growth upon CRSP8 knockdown were all significantly reversed by IKKα silencing. Similarly, IKKα overexpression also rescued the effects caused by CRSP8 overexpression (Fig. 5A–C, Supplementary Fig. S8A–C). Altogether, these results indicated that CRSP8 regulated the differentiation, EMT and cell growth by targeting IKKα in thyroid cancer.

To explore how CRSP8 negatively regulated IKKα in thyroid cancer cells, we first examined the effect of CRSP8 on the promoter (−843 to +69) activity of IKKα based on its function in transcriptional regulation. The promoter activity of IKKα was significantly increased upon CRSP8 silencing, and markedly decreased upon CRSP8 overexpression (Fig. 5D, Supplementary Fig. S8D). Furthermore, pull-down assay was performed and the binding of CRSP8 to the probe of IKKα promoter (−843 to +69) was observed, especially in ATC cells (Fig. 5E, F). In addition, CRSP8 knockdown or overexpression correspondingly decreased or increased its binding to the IKKα promoter (Fig. 5G).

To further identify the specific binding sites of CRSP8 to IKKα promoter, we carried out the dual-luciferase reporter assay with different IKKα promoter segments. CRSP8 silencing almost had no effect on the activity of IKKα promoter at the −143 to +69 sites, while significantly changed the IKKα promoter activity at the −843 to +69, −540 to +69, and −257 to +69 sites. CRSP8 overexpression caused the same effect, suggesting that CRSP8 very possibly bind to the −257 to −143 region of the IKKα promoter (Fig. 5H). ChIP assay also showed the decreased or increased binding of CRSP8 at IKKα promoter (−257 to −143) upon its knockdown or overexpression correspondingly, demonstrating again its specific binding at IKKα promoter (Fig. 5I).

Considering the classical regulation of IKKα on NF-κB pathway and the negative regulation of CRSP8 on IKKα, we then explored whether CRSP8 could regulate the NF-κB signaling. The protein levels of p-IKKα/β and p-IκBα were downregulated in CRSP8-silenced cells, but upregulated in CRSP8-overexpressed cells (Supplementary Fig. S9), suggesting CRSP8 activates NF-κB signaling in thyroid cancer cells. Combined with the results above that IKKα induced thyroid cancer cell differentiation separable from its activation on NF-κB signaling and CRSP8 dedifferentiated thyroid cancer cells via its downregulation on IKKα expression, these data implied that although CRSP8 could activate NF-κB, CRSP8 dedifferentiated thyroid cancer cell through IKKα signaling bypass NF-κB.

Given that CRSP complex is required for efficient activation by SP1, we thus proposed the synergy of SP1 in CRSP8-mediated transcriptional repression of IKKα. The significant negative correlation between CRSP8 and SP1 expression, but remarkable positive correlation between SP1 and IKKα (CHUK) expression was found based on cBioPortal database (Supplementary Fig. S10A, B). Also, compared with normal tissues, the expression of SP1 was significantly reduced in primary thyroid tumor samples (Supplementary Fig. S10C). These results suggested that CRSP8 negatively regulated IKKα expression independent of SP1.

### CRSP8 knockdown enhanced the chemosensitivity of thyroid cancer cells

**Fig. 6:**
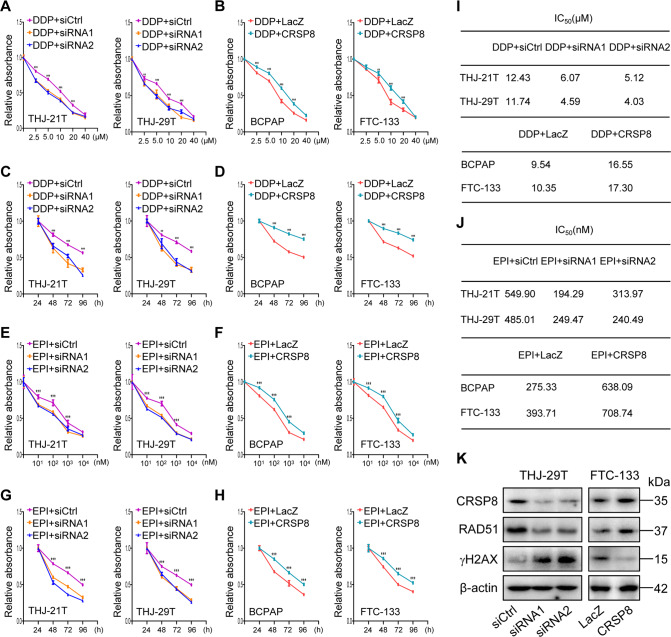
CRSP8 knockdown enhanced the antitumor effect of cisplatin and epirubicin in thyroid cancer cells. **A**–**H** The sensitivity of thyroid cancer cells to cisplatin or epirubicin treatment at different concentrations and different treatment time points were detected by MTT assay when CRSP8 was silenced or overexpressed. **I**–**J** The IC_50_ values of cisplatin and epirubicin for cell viability inhibition in cells treated with CRSP8 siRNAs or overexpression plasmids. **K** Western blot analysis of CRSP8, RAD51, and γH2AX expression in thyroid cancer cells following CRSP8 knockdown or overexpression. The data represent the mean ± SD of three independent experiments, and the level of significance was indicated by ***P < 0.001, **P < 0.01. DDP: cisplatin; EPI: epirubicin.

Accumulating evidence indicates the impairment of stemness and the enhancement of differentiation contributes to overcoming chemoresistance [27–29]. Thus, we hypothesized that CRSP8 might affect the sensitivity of thyroid cancer cells to chemotherapeutics, such as cisplatin (DDP) and epirubicin (EPI). CRSP8 knockdown increased the sensitivity of thyroid cancer cells to DDP treatment to a certain extent, while its overexpression reduced such sensitivity (Fig. 6A, B). Moreover, DDP depressed thyroid cancer cell growth in a time-dependent manner, and such depression was elevated or attenuated upon CRSP8 silencing or overexpression (Fig. 6C, D). The same phenomenon happened in thyroid cancer cells treated by EPI upon CRSP8 knockdown or overexpression (Fig. 6E–H). The IC50 values of DDP or EPI were accordingly decreased or increased when CRSP8 was silenced or overexpressed (Fig. 6I, J). These findings collectively demonstrated the contribution of CRSP8 in regulating chemosensitivity in thyroid cancer cells.

Given the cellular DNA damage functionalizes as the main effect mechanism of chemotherapy [30], we then determined the effect of CRSP8 on DNA damage. CRSP8 silencing reduced the expression of RAD51 (a marker of DNA damage repair), but increased the expression of γH2AX (a hallmark of DNA damage response), indicating that CRSP8 knockdown promoted DNA damage, thereby strengthening the cytotoxicity of chemotherapeutics, while CRSP8 overexpression caused opposite effects (Fig. 6K).

Since IKKα was negatively regulated by CRSP8, we thus investigated the contribution of IKKα in modulating chemotherapeutic sensitivity. IKKα knockdown reduced the sensitivity of thyroid cancer cells to DDP or EPI treatment, while its overexpression produced the opposite effects (Supplementary Fig. S11A–D). The IC50 values of DDP or EPI were accordingly increased or decreased when IKKα was silenced or overexpressed (Supplementary Fig. S11E, F). In agreement with CRSP8 silencing, these results indicated overexpressed IKKα restored the sensitivity of thyroid cancer cells to chemotherapy.

### CRSP8 knockdown inhibited thyroid cancer growth in vivo by upregulating IKKα

**Fig. 7:**
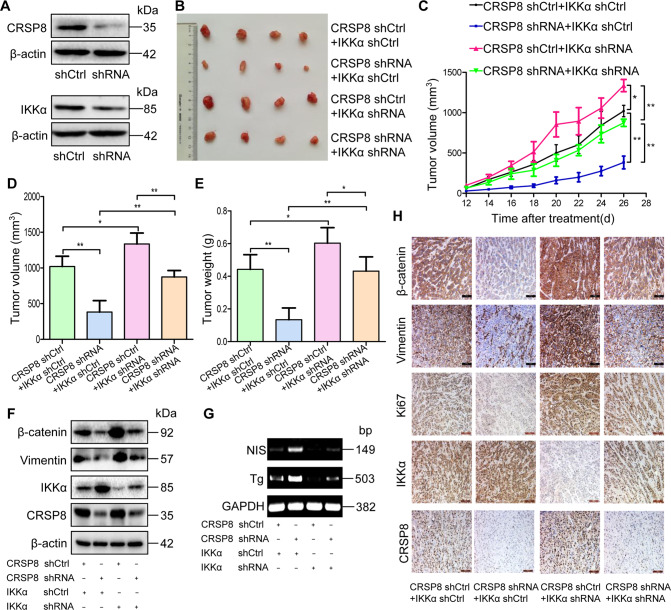
Knockdown of CRSP8 inhibited tumor growth in xenografts of thyroid cancer by upregulating IKKα expression. **A** The protein level of CRSP8 and IKKα were respectively detected in ACT-1 cells transfected with lentivirus-encapsulated scrambled shRNA or shRNA against CRSP8 or shRNA against IKKα. **B** The morphology of tumor xenografts from each nude mouse was photographed. Scale bars, 1 cm. **C** Tumor diameters of each nude mouse from different group were measured at a regular interval of 2 days after 12 days of treatment and the tumor volume was calculated as *V* = (width^2^ × length)/2. **D** Tumor volume and **E** tumor weight of nude mice from each group after sacrifice were measured. **F** The expression levels of β-catenin, Vimentin, IKKα, and CRSP8 within tumor xenografts were tested by western blot. **G** RT-PCR analysis was used to detect the expression of NIS and Tg in tumor tissues. **H** Immunohistochemistry staining of slices from xenografts was used to detect the expression of β-catenin, Vimentin, Ki67, IKKα, and CRSP8. Scale bars, 100 μm. The data represent the mean ± SD of three independent experiments, and the level of significance was indicated by **P < 0.01, *P < 0.05. *n* = 4 mice/group.

To further confirm the oncogenic role of CRSP8 in thyroid cancer progression and its dependency on IKKα signaling, we constructed thyroid cancer xenograft mouse models without or with CRSP8 knockdown or/and IKKα knockdown. The results showed that inhibition of IKKα expression effectively rescued the in vivo tumor progression delayed by CRSP8 knockdown (Fig. 7A–E, Supplementary Fig. S12A–D). Western blot and RT-PCR analysis of tumor tissues also showed that CRSP8 knockdown decreased the expression of β-catenin and Vimentin, but increased the expression of NIS and Tg, and such expression changes were rescued by IKKα knockdown (Fig. 7F, G). The IHC analyses of tumor tissues similarly revealed that CRSP8 knockdown suppressed the expression of β-catenin, Vimentin, and Ki67, but IKKα knockdown reversed such inhibition (Fig. 7H, Supplementary Fig. S12E). Together, these data confirmed that CRSP8-mediated promotion of thyroid cancer growth was realized by targeting IKKα.

## Discussion

In this study, we demonstrated the crucial role of CRSP8 in promoting thyroid cancer progression. Both thyroid cancer cells and tissues, especially ATC, embraced highly expressed CRSP8. CRSP8 knockdown decreased growth, migration, invasion and stemness, and promoted apoptosis and differentiation of thyroid cancer cells, while its overexpression induced reverse phenotypes. Mechanistically, CRSP8 transcriptionally repressed IKKα by anchoring at its specific promoter segments to dedifferentiate thyroid cancer cells. We also explored and confirmed that CRSP8 regulated the chemosensitivity of thyroid cancer cells. To the best of our knowledge, it might be the first time to report the tumor-promoting role of CRSP8 in thyroid cancer and provide novel mechanisms for such function, highlighting that CRSP8/IKKα signaling axis might be a new potential therapeutic target for differentiation therapy or combination therapy of human thyroid cancer, especially ATC.

Cancer progression is a series of dynamic molecular events leading to genetic instability and aberrant phenotype [31]. The distinctive feature of disease progression is cellular dedifferentiation [32–34]. During thyroid cancer progression, cellular dedifferentiation occurs in ~5% of cases and is usually accompanied by more aggressive growth, metastatic spread, and loss of iodide uptake ability, making the tumor resistant to the conventional treatments and radioiodine therapy [35–37]. Considering that differentiation level determined the malignancy of thyroid cancer, and CRSP8 expression was negatively correlated with the differentiation level of thyroid cancer, we deduced CRSP8 might promote thyroid cancer progression by inducing dedifferentiation. In agreement with our hypothesis, overexpressed CRSP8 in DTC cells led to the obvious changes at protein levels and functions associated with dedifferentiation, including promoted growth, metastasis and stem-like properties and decreased expression of NIS and Tg. Accordingly, the significant changes at protein levels and functions associated with differentiation induction happened upon CRSP8 silencing in ATC cells. Notably, besides CRSP8, IKKα, a part of the IκB kinase complex known to regulate the NF-κB signaling [38], was similarly found to regulate thyroid cancer cell differentiation. Overexpressed IKKα can reverse the poorly differentiated state, as evidenced by the elevated expression of NIS and Tg in ATC cells. Combined with the previous reports that IKKα regulated epidermal differentiation [39, 40], induced nasopharyngeal carcinoma differentiation and reduced its tumorigenicity [11], our findings highlighted that IKKα might be a pivotal marker for predicting the differentiation status of thyroid cancer cells. Intriguingly, our data also indicated the differentiation regulation by IKKα in thyroid cancer was independent of NF-κB signaling, which was consistent with the previous reports that IKKα induced keratinocyte terminal differentiation [41] and articular chondrocyte differentiation [42] independent of NF-κB. Identifying and validating the precise pathway activated by IKKα in its regulation on thyroid cancer cell differentiation will be a focus of our ongoing work.

Since CRSP8 and IKKα both regulated thyroid cancer cell differentiation, we next explored the potential relationship between them. As expected, IKKα expression was strictly controlled by CRSP8, and IKKα knockdown or overexpression significantly reversed CRSP8 knockdown or overexpression-mediated promotion or suppression of thyroid cancer cell differentiation. Similarly, the inhibition of tumor growth caused by CRSP8 knockdown was rescued by IKKα knockdown, confirming the significance of IKKα as the key downstream target of CRSP8 to regulate thyroid cancer cell differentiation and survival. Moreover, we provided mechanistic insights into CRSP8 regulating IKKα expression. Considering CRSP8 itself is a transcriptional coactivator, we hypothesized its direct transcriptional regulation on IKKα and experimentally verified this, as evidenced by its anchoring at IKKα promoter regions to negatively regulate IKKα promoter activity. The precise anchoring site was also identified and confirmed via different IKKα promoter regions-driven luciferase reporter assay and ChIP analysis. Besides, as a subunit of CRSP complex which is required for efficient activation by SP1, CRSP8 was therefore reasonably inferred to synergize with SP1 to negatively regulate IKKα. Nevertheless, the remarkable positive correlation between SP1 and IKKα expression, and the significant negative correlation between SP1 and CRSP8 expression in thyroid cancer denied such possibility. Hence, since SP1 does not seem work, what are the transcriptional factors exactly recruited by CRSP8 in achieving its control on IKKα transcription? Do they function as activators or suppressors in initiating IKKα transcription? All these questions deserve further investigations.

Combined with the newly report that DNA damage activated IKKα [43], and our findings that CRSP8 knockdown resulted in DNA damage, we do not exclude the possibility of indirect regulation that CRSP8 knockdown causes DNA damage thus activates IKKα, just this activation is reflected by the rapid induction of p-IKKα without any change of the total IKKα expression according to the report, which is inconsistent with our results that CRSP8 knockdown increased total IKKα expression, but not p-IKKα. Thus, compared with the direct transcriptional regulation approved in our study, such indirect regulation might still need further exploration. Besides, CRSP8 was recently reported to be reversely regulated by miR-18a, which belongs to the miR-17-92 cluster, as its direct target in osteosarcoma [44], implying the possibility that miR-18a might similarly regulate CRSP8 in thyroid cancer cells. Nevertheless, considering earlier reports that all members of miR-17-92 cluster except miR-17-3p were overexpressed and partial play oncogenic function in ATC [45], the hypothesis mentioned above seems not valid. Of course, whether CRSP8/IKKα signaling is under control of microRNA is still inconclusive and deserves better study.

On the basis of the experimental and clinical observations, together with mechanistic findings, our research has demonstrated the oncogenic role of CRSP8 in thyroid cancer progression depending on its reverse transcriptional regulation on IKKα expression, and proposed the therapeutic strategy of targeting CRSP8-mediated dedifferentiation in human thyroid cancer with high malignancy. Although more in-depth mechanisms, the diagnostic and prognostic roles for CRSP8 in thyroid cancer need further investigation, our findings at least suggest that silencing CRSP8 expression or blocking its binding to IKKα promoter or activating IKKα expression can ameliorate the outcome of patients with thyroid cancer, especially the ones with ATC, by promoting tumor differentiation, limiting tumor metastasis and recurrence.

## Supplementary information

Supplementary figure legends

Supplementary Figure 1

Supplementary Figure 2

Supplementary Figure 3

Supplementary Figure 4

Supplementary Figure 5

Supplementary Figure 6

Supplementary Figure 7

Supplementary Figure 8

Supplementary Figure 9

Supplementary Figure 10

Supplementary Figure 11

Supplementary Figure 12
