# Supplementary material for: CRSP8 promotes thyroid cancer progression by antagonizing IKKα-induced cell differentiation
Source: Cell Death Differ. 2020 Nov 8;28(4):1347–63. doi: 10.1038/s41418-020-00656-0 (PMC8027816; doi:10.1038/s41418-020-00656-0)
Supplement: Supplementary file 1 — Supplementary figure legends [file 41418_2020_656_MOESM1_ESM.docx]

**Supplementary Figure legends**

**Fig. S1 Expression of CRSP8 in human thyroid cancer.**

(A) Expression of CRSP8 in primary thyroid cancer samples compared with normal samples determined by the UALCAN analysis. (B) CRSP8 expression at mRNA level in thyroid cancer analyzed from Oncomine database. No value was used as control. (C) UALCAN analysis of CRSP8 expression in thyroid cancer based on histological subtype. (D) Microarray gene expression data from GEO data set GSE53072 assessed the expression of CRSP8 in normal and anaplastic thyroid carcinomas. The level of significance was indicated by ***P < 0.001, **P < 0.01, *P < 0.05.

**Fig. S2 PI3K/AKT signaling pathway was involved in the growth regulation of thyroid cancer cells mediated by CRSP8.**

(A, B) THJ-29T and FTC-133 cells were pre-treated with LY294002 (50 μM) for 4 h, followed by the transfection with CRSP8 specific siRNAs or overexpression plasmids. After 48 h, MTT assay was used to detect cell viability. The data represent the mean ± SD of three independent experiments, and the level of significance was indicated by ***P < 0.001, **P < 0.01, ns: non-significant (p > 0.05). LY: LY294002.

**Fig. S3 CRSP8 regulated apoptosis and autophagy of thyroid cancer cells.**

(A) FACS analysis was performed to detect the apoptotic level of tumor cells. THJ-21T and THJ-29T cells pretreated with DMSO or Z-VAD (20 μM) for 4 h were transfected with CRSP8 specific siRNA or control siRNA. After 48 h, cells were harvested, dually labeled with Annexin V and PI, and subjected to FACS analysis. (B) The percentage of apoptotic cells in (A) was assessed. (C) Western blot analysis for the expression of autophagy-related proteins LC3-I/II and p62 in thyroid cancer cells following CRSP8 knockdown or overexpression. The data represent the mean ± SD of three independent experiments, and the level of significance was indicated by ***P < 0.001, **P < 0.01, ns: non-significant (p > 0.05).

**Fig. S4 CRSP8 promoted the viability and invasion of thyroid cancer cells.**

(A) Confirmation of CRSP8 knockdown (siCRSP8), re-expression (siRES) in thyroid cancer cell lines by western blot analysis. (B, C) The effects of CRSP8 knockdown or re-expression on viability (B) and invasion (C) of thyroid cancer cells were respectively analyzed. The number of invading cells was counted (right of C). Scale bars, 200 μm. The data represent the mean ± SD of three independent experiments, and the level of significance was indicated by ***P < 0.001, **P < 0.01, ns: non-significant (p > 0.05).

**Fig. S5** **CRSP8 was involved in the differentiation and stemness regulation of thyroid cancer cells.**

(A) Gene set enrichment analysis associated with CRSP8 expression. The gene set of epidermal cell differentiation pathway was enriched in thyroid cancer samples with low expression of CRSP8. (B) RT-PCR analysis of CRSP8, NIS and Tg expression in thyroid cancer cells transfected with CRSP8 specific siRNAs or its overexpression plasmids. (C) The representative images of tumor sphere formation in thyroid cancer cells with CRSP8 silencing or overexpression. The number of spheres bigger than 50 µm were quantified and shown. Scale bars, 200 µm. (D) The expression of stemness-associated markers, including CD133 and CD44 were determined by Western blot following CRSP8 knockdown or overexpression. The data represent the mean ± SD of three independent experiments, and the level of significance was indicated by ***P < 0.001, **P < 0.01.

**Fig. S6 IKKα induced differentiation of thyroid cancer cells independent of NF-κB signaling activation.**

(A) RT-PCR analysis of IKKα, NIS and Tg expression in thyroid cancer cells transfected with IKKα specific siRNAs or its overexpression plasmids. (B) The subcellular localization of p65/p50 following IKKα knockdown or overexpression was tested by immunofluorescence. (C) The expression of p65/p50 in the nucleus was detected by western blot assay in BCPAP and THJ-29T cells with IKKα knockdown or overexpression. LAMB1 was served as the loading control. (D) Protein levels of phospho-p65 and total p65 in thyroid cancer cells treated with indicated concentrations of QNZ or JSH-23 were analyzed by Western blot. (E) THJ-21T and THJ-29T cells pre-treated with NF-κB inhibitors QNZ (10 μM) or JSH-23 (20 μM) were transfected with plasmids carrying IKKα gene or control gene (LacZ). The expression of IKKα, NIS and Tg were measured by Western blot. (F) BCPAP and FTC-133 cells pre-treated with a pan-caspase inhibitor Z-VAD (20 μM) were transfected with IKKα specific siRNA or control siRNA. The expression of IKKα, NIS and Tg were measured by Western blot. NC: control siRNA.

**Fig. S7 Negative correlation between CRSP8 and** **IKKα expression** **in thyroid cancer cells.**

Western blot analysis of IKKα, CRSP8, NIS and Tg expression in thyroid cancer cells with different differentiation degree.

**Fig. S8 CRSP8 knockdown promoted the differentiation of thyroid cancer cells by transcriptionally up-regulating IKKα expression.**

(A, B) The expression of NIS and Tg were detected by RT-PCR in thyroid cancer cells with indicated treatment. (C) CRSP8 was silenced in THJ-29T cells or overexpressed in BCPAP cells and then treated with IKKα siRNA or plasmids in these two cells, respectively. Cell viability was measured by MTT assay. (D) Dual-luciferase reporter assay was performed to determine relative IKKα promoter activity following CRSP8 knockdown or overexpression. The data represent the mean±SD of three independent experiments, and the level of significance was indicated by ***P < 0.001, **P < 0.01.

**Fig. S9** **CRSP8 itself activated the NF-κB signaling pathway in thyroid cancer cells.**

Western blot analysis of the expression of NF-κB signaling pathway-related proteins in thyroid cancer cells following CRSP8 knockdown or overexpression.

**Fig. S10 The expression analysis of SP1 in thyroid cancer and its expression correlation with CRSP8 and IKKα.**

(A, B) The expression correlation of CRSP8 and SP1, SP1 and CHUK (IKKα) were respectively detected using cBioPortal database, n=509. (C) Expression of SP1 in primary thyroid cancer samples compared with normal samples determined by the UALCAN analysis.

**Fig. S11 Overexpression of** **IKKα sensitized thyroid cancer cells to chemotherapeutic drugs.**

(A-D) Thyroid cancer cells were transfected with IKKα specific siRNAs or its overexpression plasmids, and then treated with different concentrations of DDP or EPI. Cell viability was measured using MTT analysis. (E, F) The IC50 values of DDP or EPI in thyroid cancer cells with IKKα knockdown or overexpression were calculated respectively. The data represent the mean±SD of three independent experiments, and the level of significance was indicated by ***P < 0.001, **P < 0.01, *P < 0.05. DDP: cisplatin; EPI: epirubicin.

**Fig. S12 Knockdown of CRSP8 suppressed tumor growth in vivo by up-regulating the expression of** **IKKα.**

(A) The morphology of tumor xenografts from each nude mouse was photographed. Scale bars, 1cm. n = 5 mice/group. (B) Tumor diameters of each nude mouse from different group were measured at a regular interval of 3 days after 9 times of injection and the tumor volume was calculated as V= (width^2^×length)/2. (C) Tumor volume and (D) tumor weight of nude mice from each group after sacrifice were measured. (E) Immunohistochemistry staining of slices from tumor tissues was used to detect the expression of β-catenin, Vimentin, Ki67, IKKα, and CRSP8. The data represent the mean±SD of three independent experiments, and the level of significance was indicated by ***P < 0.001, **P < 0.01.
