# Supplementary figures and images for: CRSP8 promotes thyroid cancer progression by antagonizing IKKα-induced cell differentiation
Source: Cell Death Differ. 2020 Nov 8;28(4):1347–63. doi: 10.1038/s41418-020-00656-0 (PMC8027816; doi:10.1038/s41418-020-00656-0)

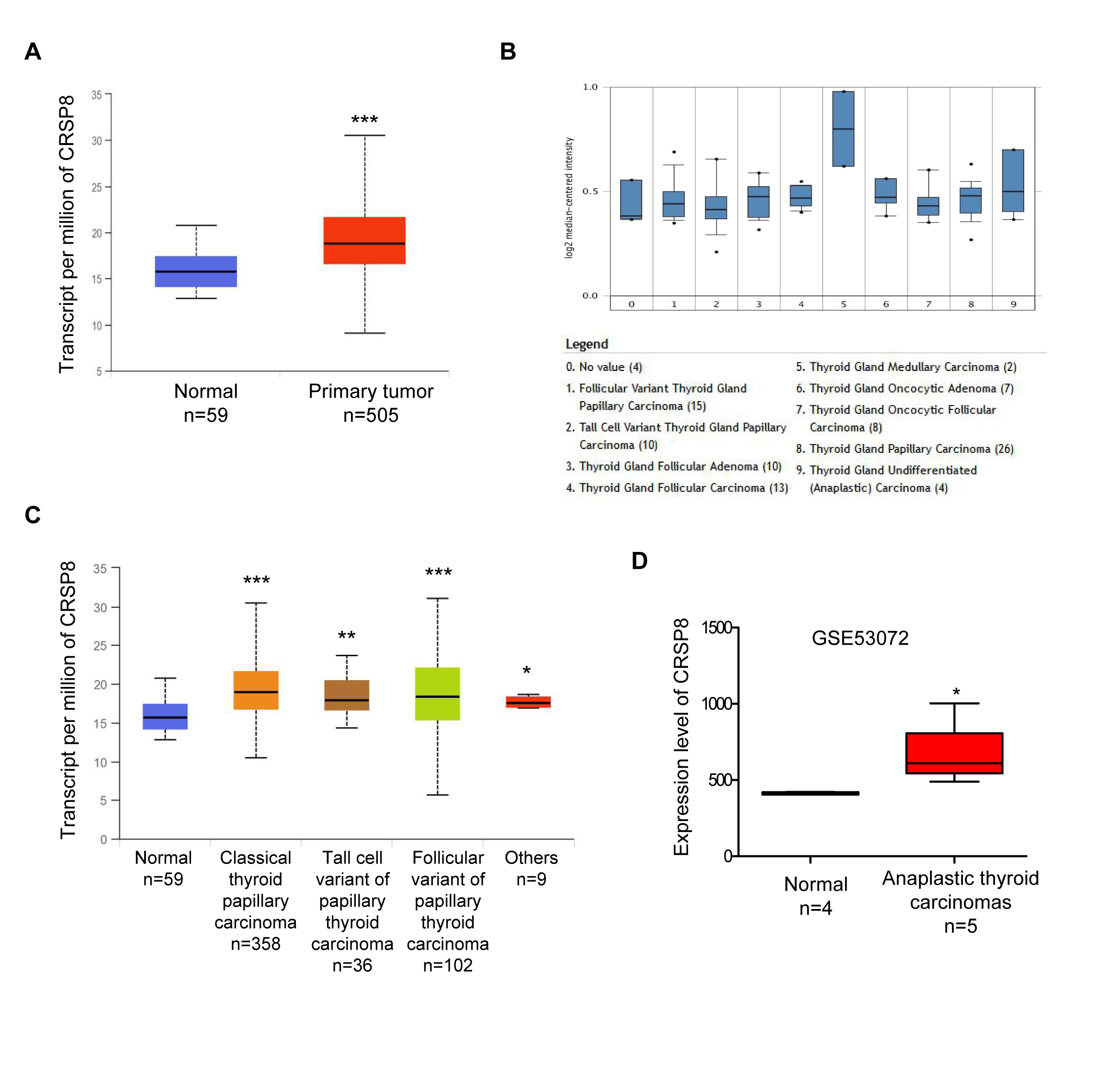

Supplement: Supplementary file 2 — Supplementary Figure 1 [file 41418_2020_656_MOESM2_ESM.tif]

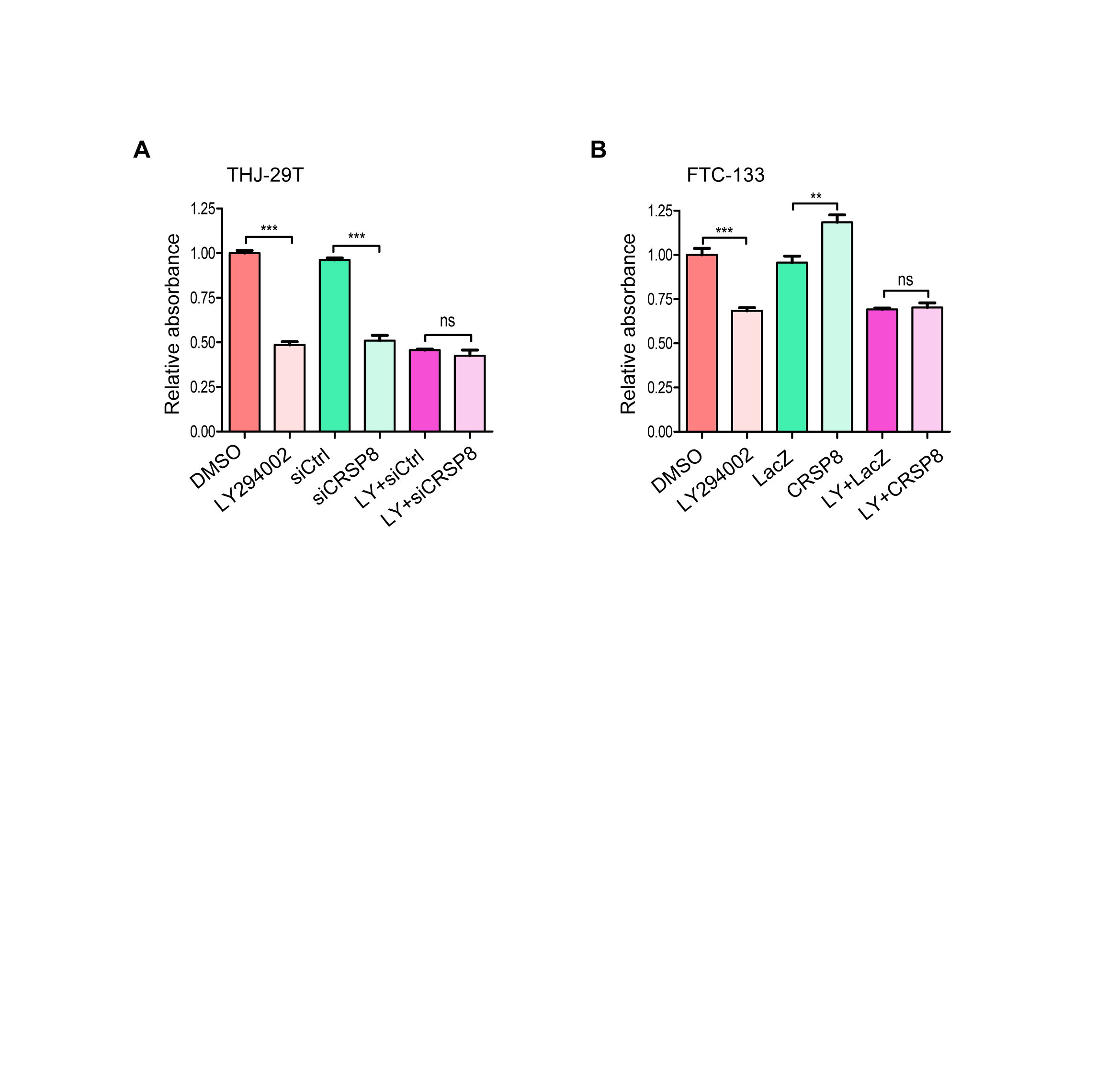

Supplement: Supplementary file 3 — Supplementary Figure 2 [file 41418_2020_656_MOESM3_ESM.tif]

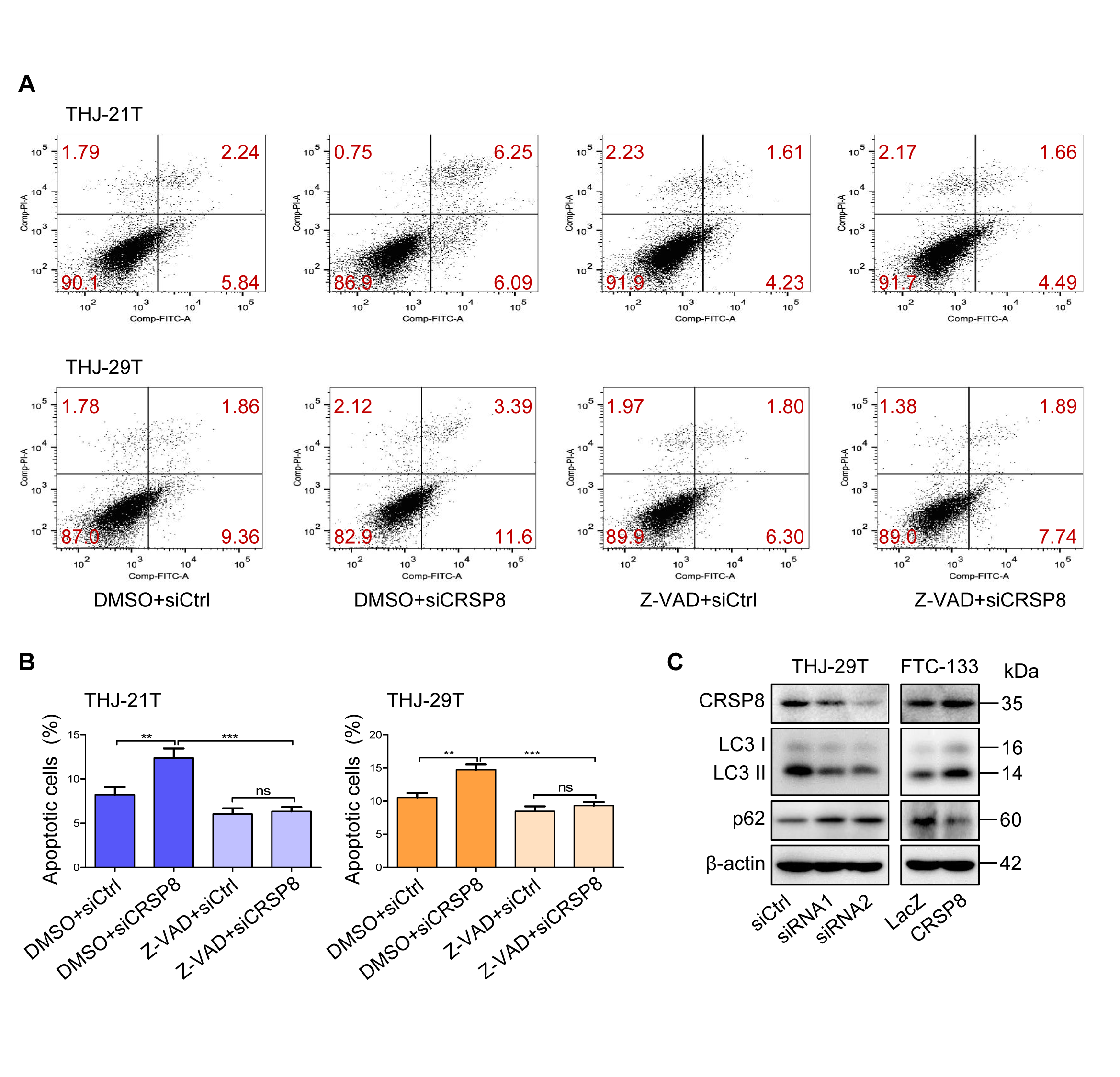

Supplement: Supplementary file 4 — Supplementary Figure 3 [file 41418_2020_656_MOESM4_ESM.tif]

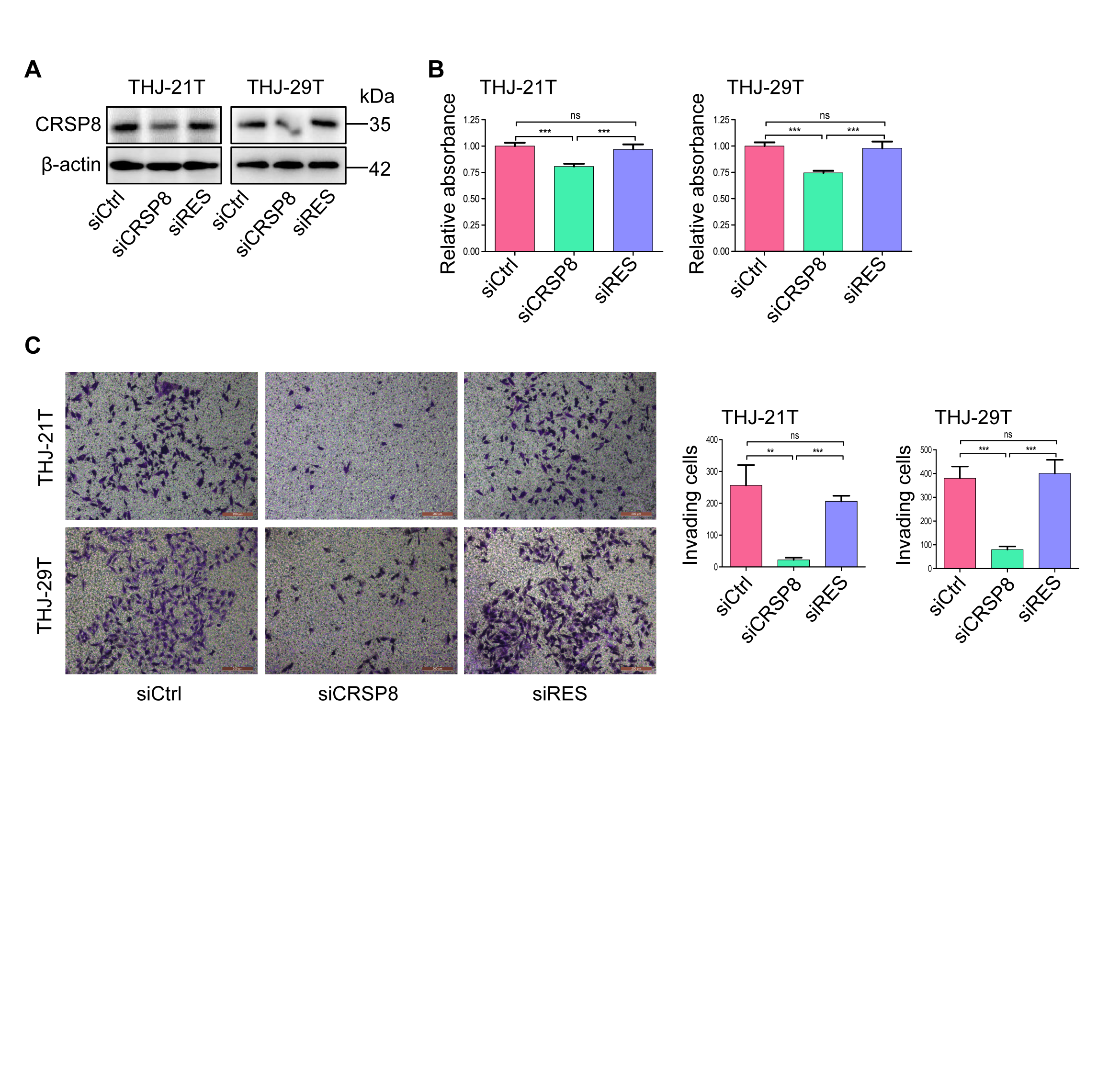

Supplement: Supplementary file 5 — Supplementary Figure 4 [file 41418_2020_656_MOESM5_ESM.tif]

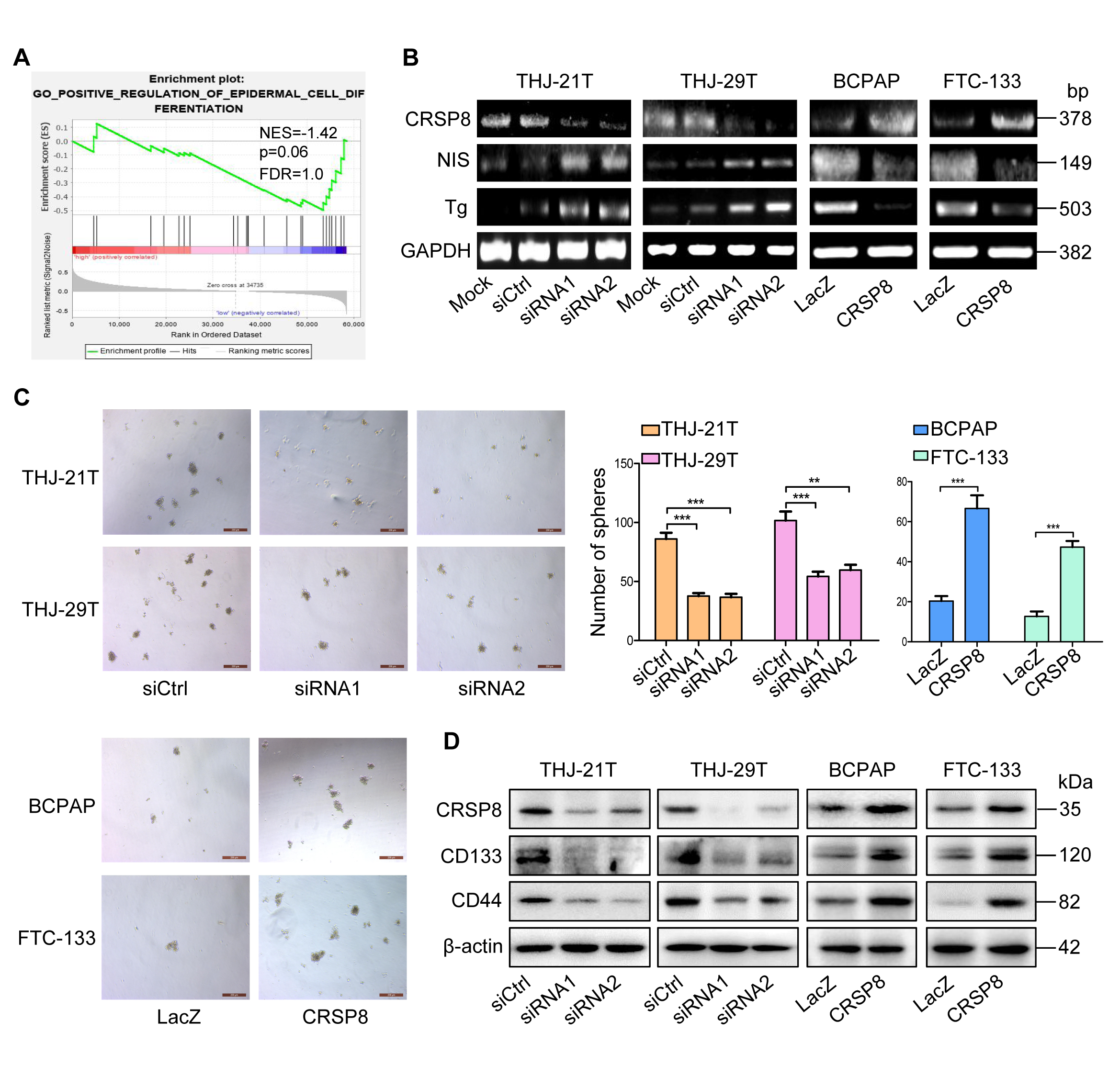

Supplement: Supplementary file 6 — Supplementary Figure 5 [file 41418_2020_656_MOESM6_ESM.tif]

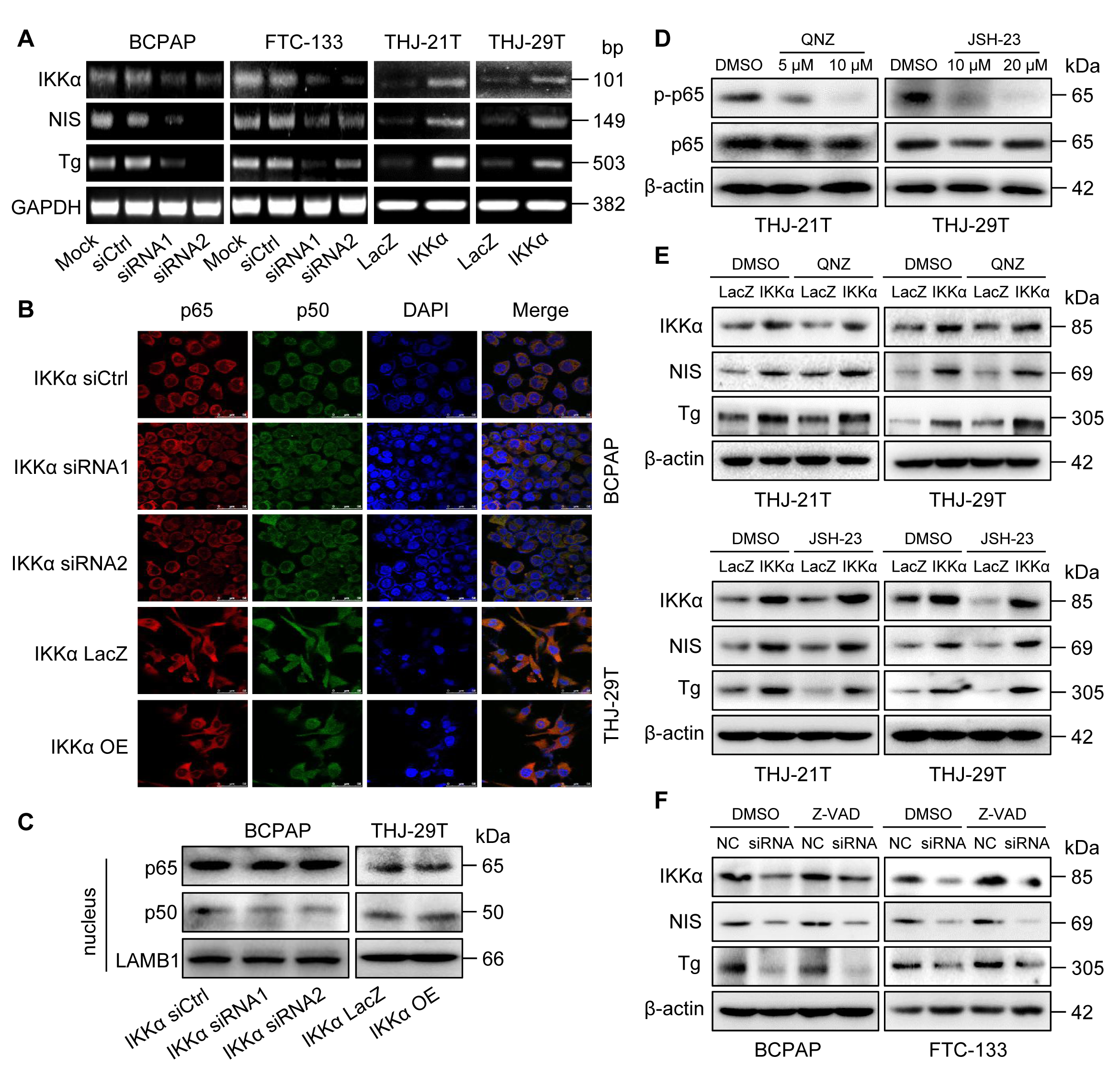

Supplement: Supplementary file 7 — Supplementary Figure 6 [file 41418_2020_656_MOESM7_ESM.tif]

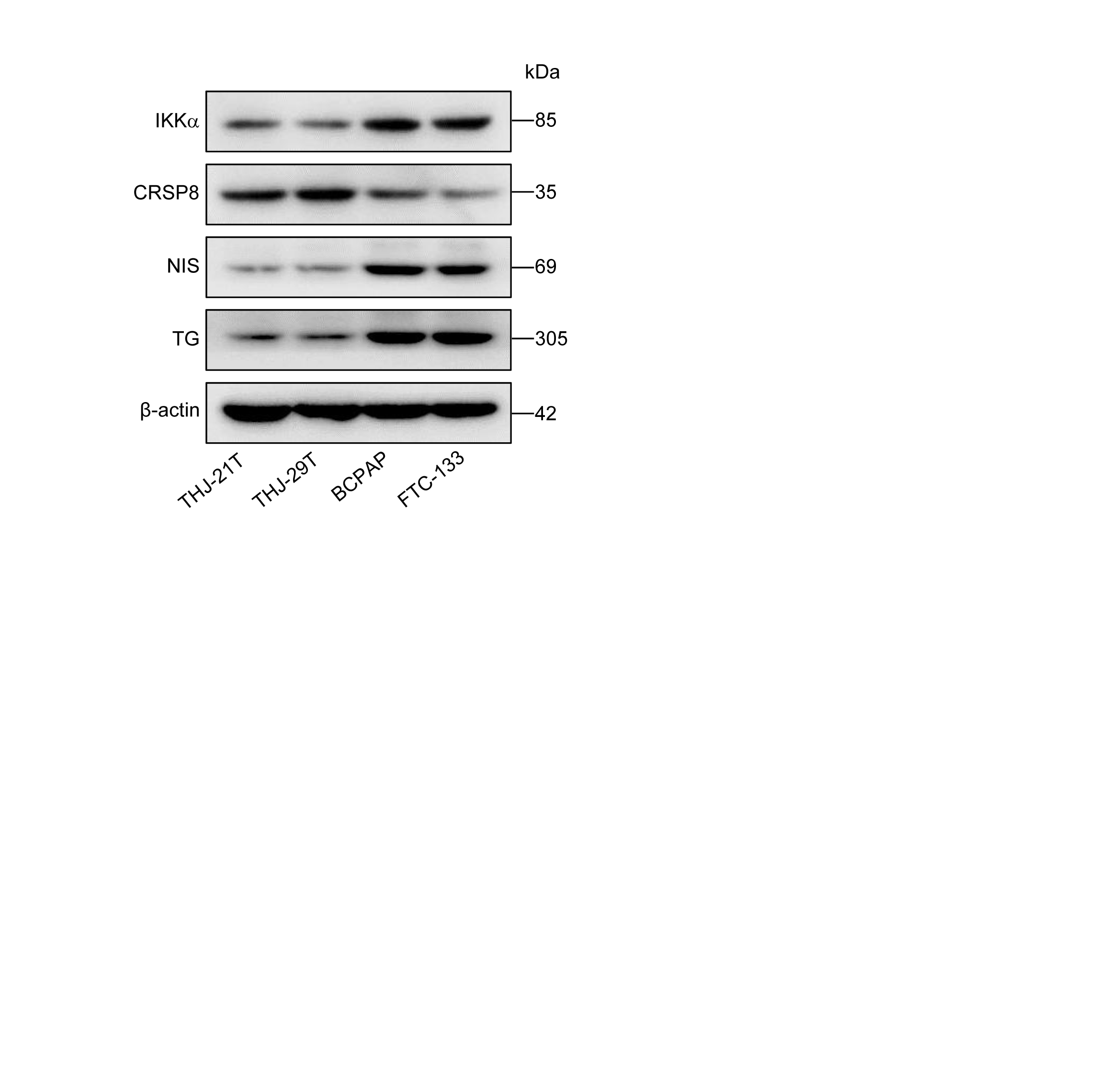

Supplement: Supplementary file 8 — Supplementary Figure 7 [file 41418_2020_656_MOESM8_ESM.tif]

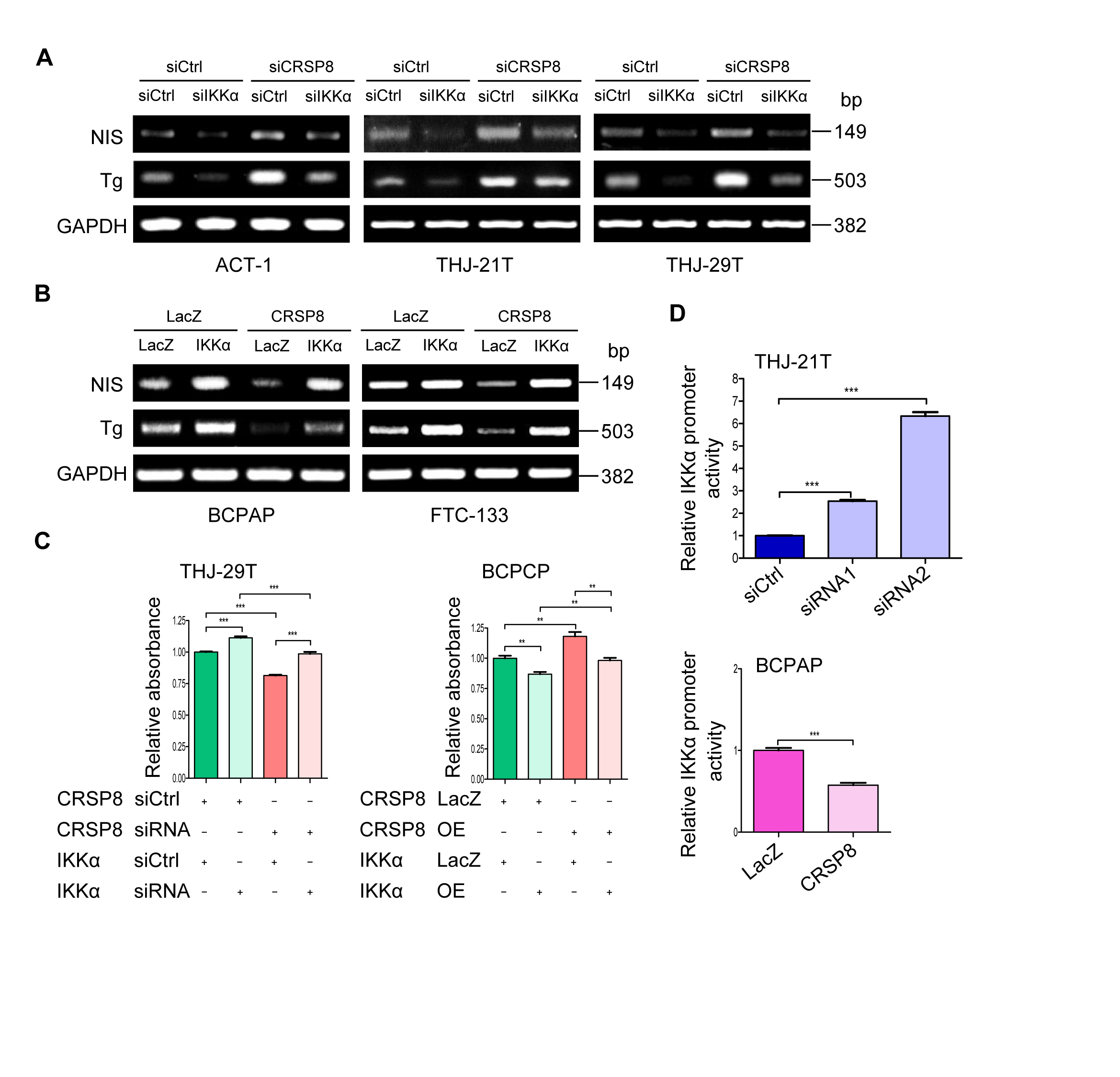

Supplement: Supplementary file 9 — Supplementary Figure 8 [file 41418_2020_656_MOESM9_ESM.tif]

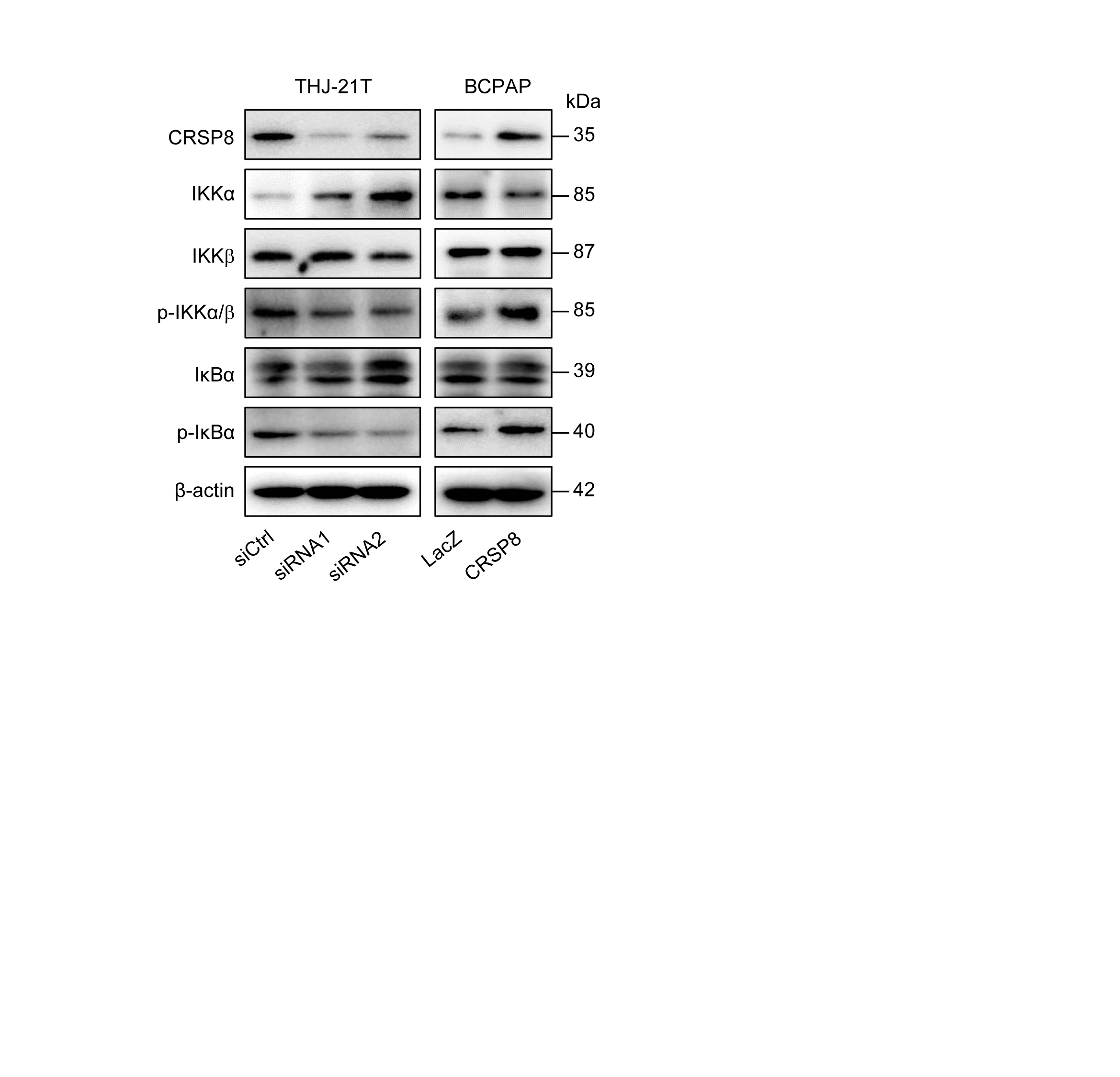

Supplement: Supplementary file 10 — Supplementary Figure 9 [file 41418_2020_656_MOESM10_ESM.tif]

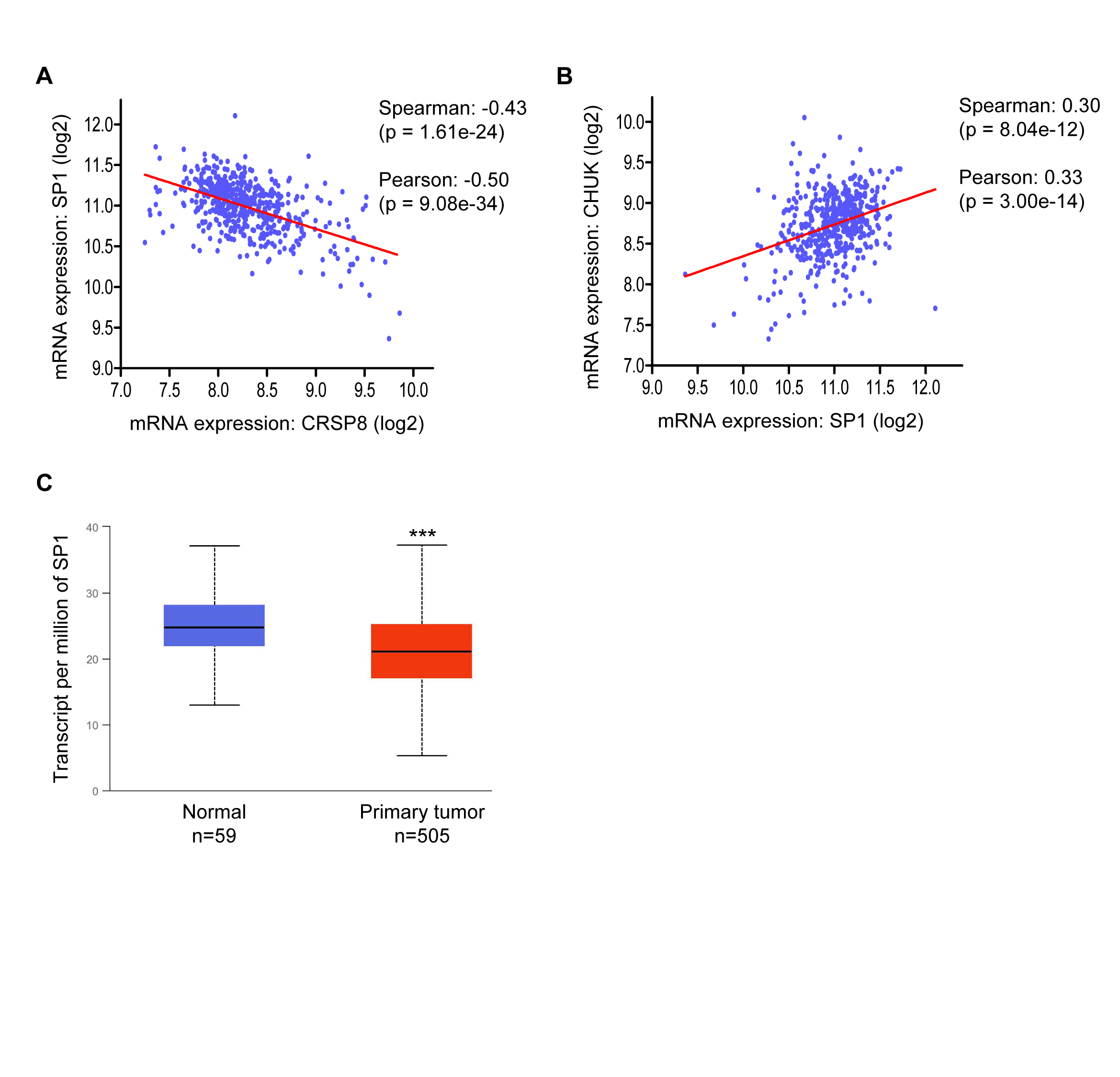

Supplement: Supplementary file 11 — Supplementary Figure 10 [file 41418_2020_656_MOESM11_ESM.tif]

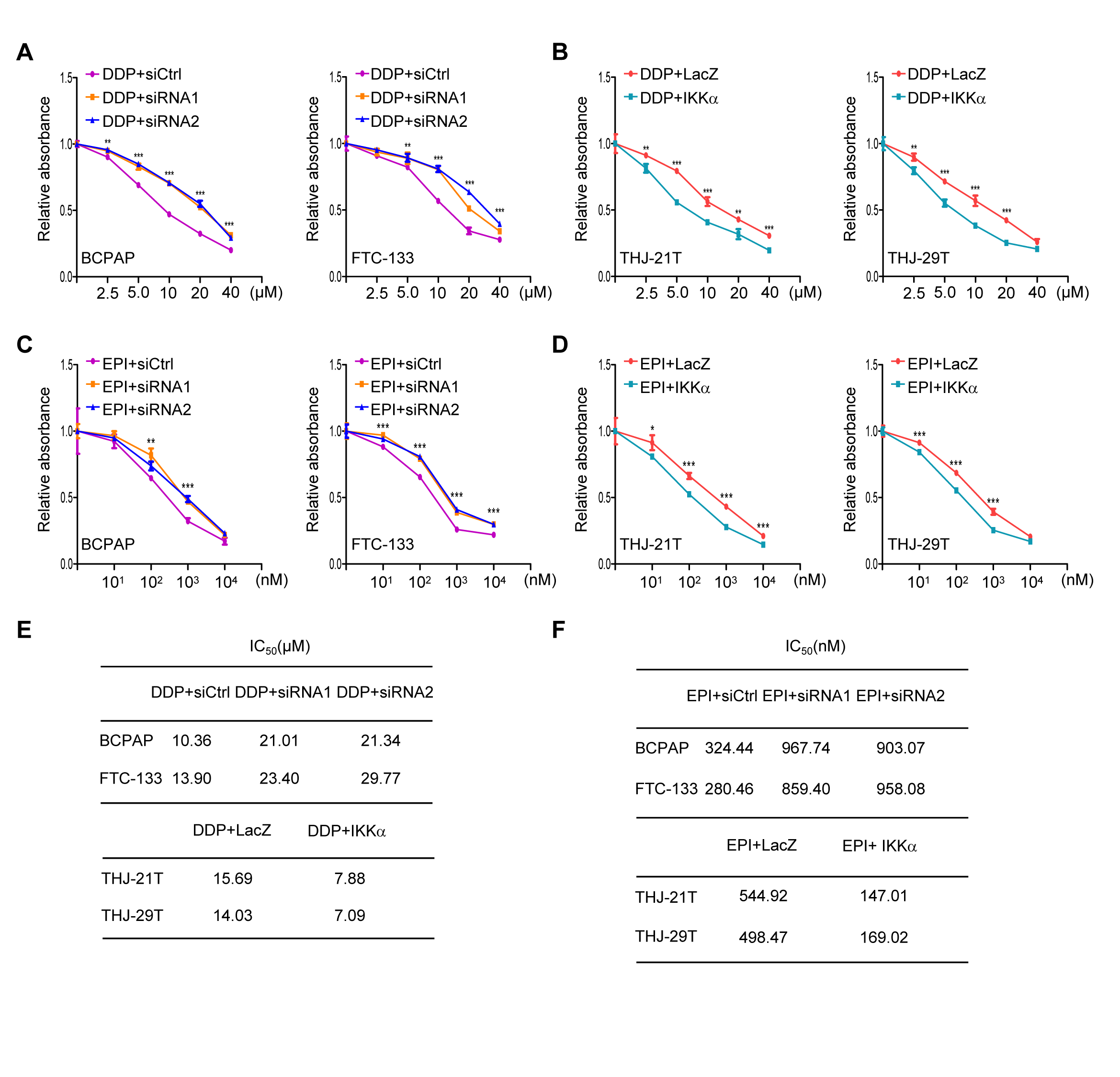

Supplement: Supplementary file 12 — Supplementary Figure 11 [file 41418_2020_656_MOESM12_ESM.tif]

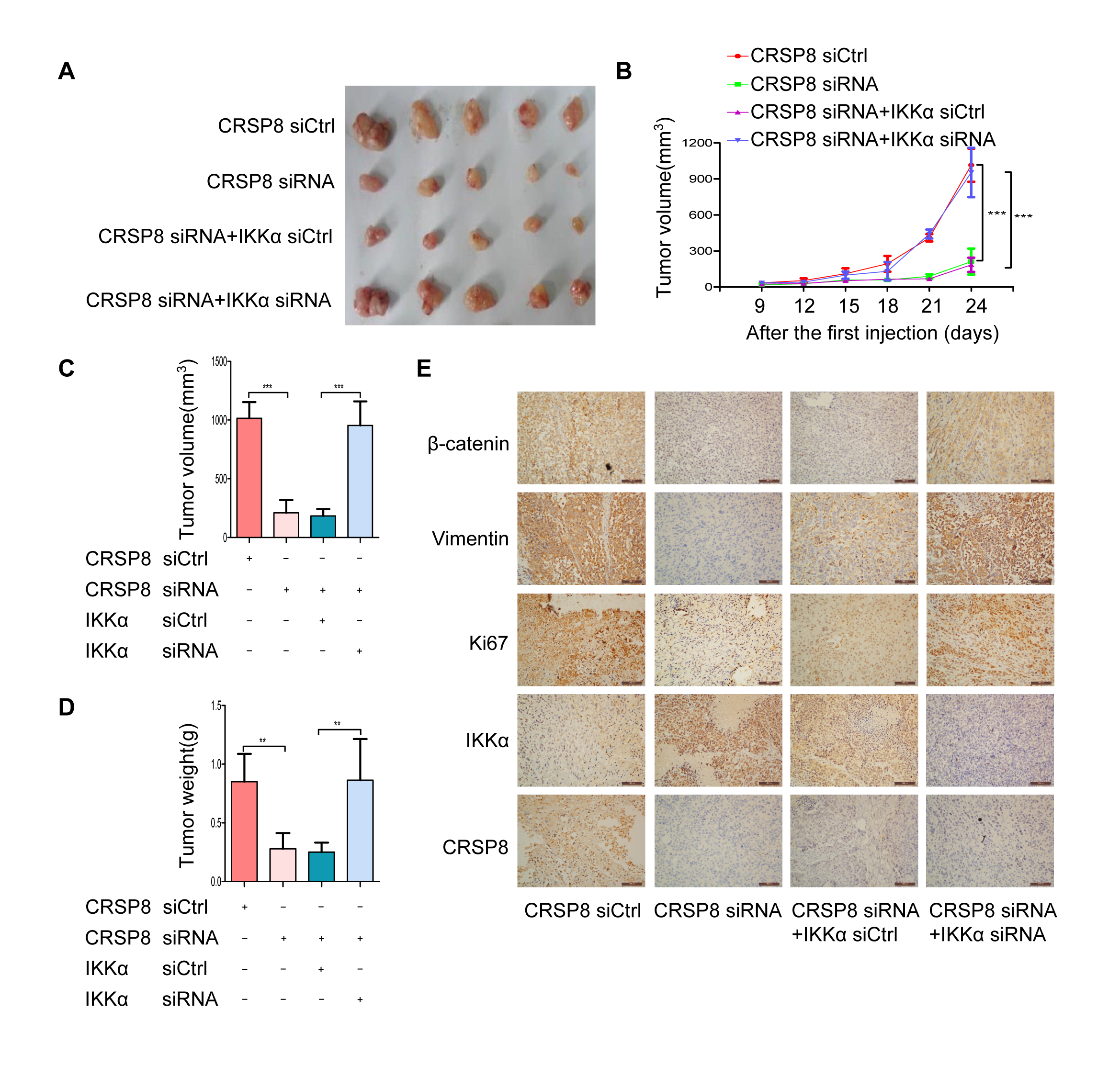

Supplement: Supplementary file 13 — Supplementary Figure 12 [file 41418_2020_656_MOESM13_ESM.tif]
